# On the ecological impact of prehistoric hunter-gatherers in Europe: Early Holocene (Mesolithic) and Last Interglacial (Neanderthal) foragers compared

**DOI:** 10.1371/journal.pone.0328218

**Published:** 2025-10-22

**Authors:** Anastasia Nikulina, Anhelina Zapolska, Maria Antonia Serge, Didier M. Roche, Florence Mazier, Marco Davoli, Elena A. Pearce, Jens-Christian Svenning, Dave van Wees, Ralph Fyfe, Katharine MacDonald, Wil Roebroeks, Fulco Scherjon

**Affiliations:** 1 Faculty of Archaeology, Leiden University, Leiden, The Netherlands; 2 Department of Archaeology, Durham University, Durham, United Kingdom; 3 Earth and Climate Cluster, Faculty of Sciences, Vrije Universiteit Amsterdam, Amsterdam, The Netherlands; 4 Laboratoire Géographie de l’Environnement, GEODE UMR, Université de Toulouse-Jean Jaurès, Toulouse, France; 5 Laboratoire des Sciences du Climat et de l’Environnement, LSCE/IPSL, CEA-CNRS-UVSQ, Université Paris-Saclay, Gif-sur-Yvette, France; 6 Center for Ecological Dynamics in a Novel Biosphere (ECONOVO), Department of Biology, Aarhus University, Aarhus, Denmark; 7 Department of Biology and Biotechnologies, Sapienza University of Rome, Rome, Italy; 8 BeZero Carbon Ltd., London, United Kingdom; 9 School of Geography, Earth and Environmental Sciences, University of Plymouth, Plymouth, United Kingdom; 10 MONREPOS Archaeological Research Centre and Museum for Human Behavioural Evolution, Neuwied, Germany; University of California Santa Cruz, UNITED STATES OF AMERICA

## Abstract

Recent studies have highlighted evidence of human impact on landscapes dating back to the Late Pleistocene–long before the advent of agriculture. Quantifying the extent of vegetation transformations by hunter-gatherers remains a major research challenge. We address this challenge by comparing climate-based potential natural vegetation cover with pollen-based vegetation reconstructions for the Last Interglacial and the Early Holocene. Differences between these datasets suggest that climate alone cannot fully explain the pollen-based vegetation patterns in Europe during these periods. To explore this issue, we used an upgraded version of the HUMan impact on LANDscapes (HUMLAND) agent-based model (ABM), combined with a genetic algorithm, to generate vegetation change scenarios. By comparing ABM outputs with pollen-based reconstructions, we aimed to identify parameter values that yield HUMLAND results closely matching the pollen-based vegetation cover. The updated ABM covers a broad temporal range, and incorporates the effects of hunting on herbivores and their influence on vegetation regeneration. The results show that the combined effects of megafauna, natural fires, and climatic fluctuations alone lead to vegetation cover estimates that are inconsistent with paleoecological reconstructions. Instead, anthropogenic burning played a key role, with modelling results suggesting that European landscapes were already substantially modified by humans by the Early Holocene. In scenarios where human-induced burning was minimal or absent, foragers still shaped landscapes indirectly through hunting, which influenced herbivore densities and their impact on vegetation dynamics. Our study revealed that Neanderthals and Mesolithic humans influenced similar-sized areas around their campsites and shared comparable preferences for vegetation openness. Our results challenge the assumption that pre-agricultural humans had minimal ecological impact. Instead, this study provides strong evidence that both Neanderthals and Mesolithic foragers actively shaped European interglacial ecosystems, influencing vegetation dynamics long before agriculture.

## Introduction

The past relationships between humans and their environment have been the subject of extensive research. While the emergence of agriculture is commonly regarded as the starting point for a strong anthropogenic influence on vegetation cover, recent studies have highlighted the substantial impact of hunter-gatherer communities on their environment through repetitive burning of vegetation [1–12]. It is important to recognize and assess the long-term effects of these early human activities preceding the emergence of agriculture [5]. Biodiversity conservation efforts often require a reference ecosystem or baseline [13], an inferred natural state before large-scale human exploitation of resources [14]. Identifying such baselines is challenging due to the complexities of past environmental processes [15]. Thus, studying the impact of early human activities on their environment is crucial not only for archaeology and related fields but also for informing ecosystem restoration projects aimed at a sustainable future.

**Fig 1:**
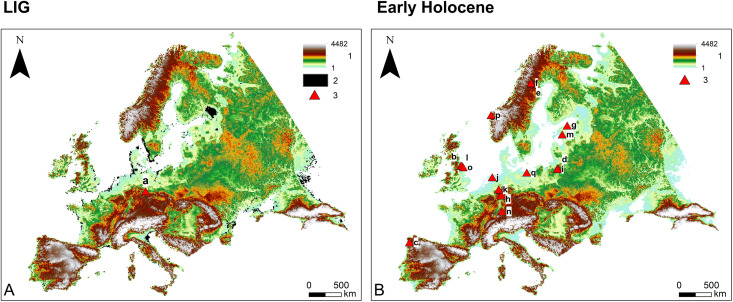
LIG (A) and Early Holocene (B) study area. Legend: 1–Elevations (in meters above sea level, m a.s.l.); 2–No data; 3–Case studies indicating possible vegetation burning by LIG and Early–Middle Holocene hunter-gatherers [4,9–12,16,17]. List of case studies: a–Neumark-Nord; b–Bonfield Gill Head; c–Campo Lameiro; d–Dudka Island; e–Dumpokjauratj; f–Ipmatisjauratj; g–Kunda-Arusoo; h–Lahn valley complex; i–Lake Miłkowskie; j–Meerstad; k–Mesolithic site at Soest; l–North Gill; m–Pulli; n–Rottenburg-Siebenlinden sites; o–Star Carr; p–Vingen sites; q–Wolin II.

**Fig 2:**
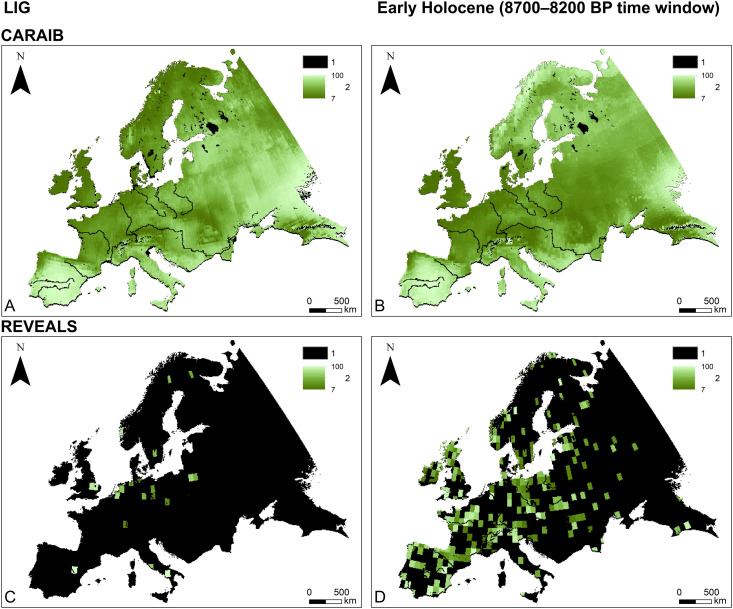
Vegetation openness: CARbon Assimilation In the Biosphere (CARAIB) LIG (A), CARAIB 8700–8200 BP (B); Regional Estimates of VEgetation Abundance from Large Sites (REVEALS) mesocratic I (C), REVEALS 8700–8200 BP. Vegetation openness for other time windows available in Supporting Information (S1 and S2 Figs S1 File.). Legend: 1–No data; 2–Vegetation openness (%).

**Fig 3:**
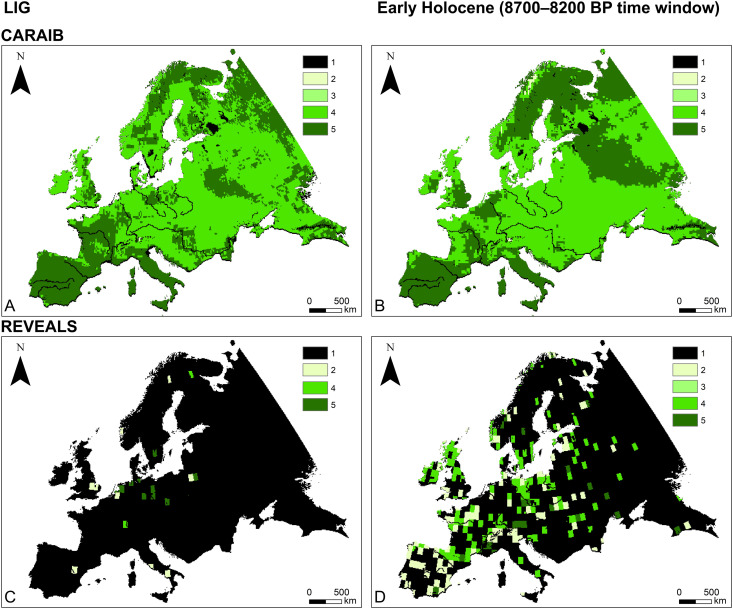
Distribution of dominant plant functional types (PFTs): CARAIB LIG (A), CARAIB 8700–8200 BP (B); REVEALS mesocratic I (C), REVEALS 8700–8200 BP. PFT distribution for other time windows available in Supporting Information (S3 and S4 Figs S1 File.). Legend: 1–No data; 2–Herbs; 3–Shrubs; 4–Broadleaf trees; 5–Needleleaf trees.

**Fig 4:**
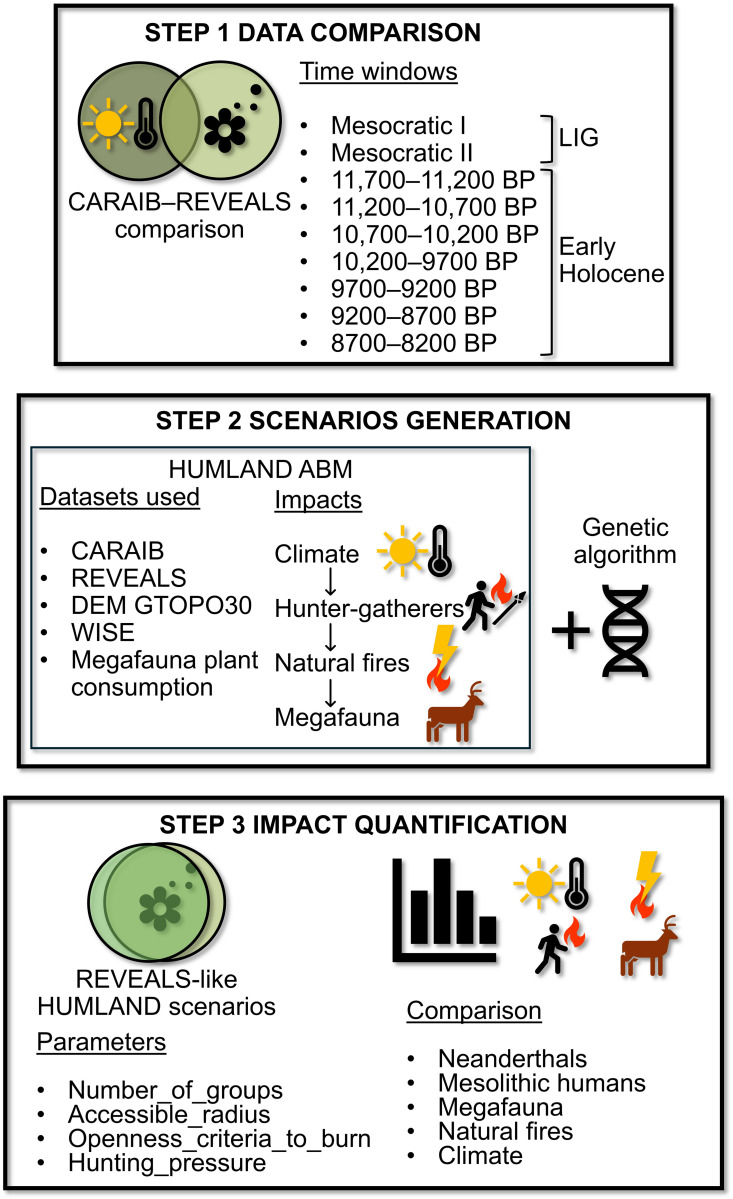
Overview of research steps including the comparison of CARAIB (climate-driven potential natural vegetation) and REVEALS (pollen-based vegetation reconstruction) data, the development and upgrade of the HUMLAND ABM, its integration with a genetic algorithm, and the generation of scenarios to quantify the impacts of Neanderthals, Mesolithic population, megafauna, natural fires, and climate on vegetation.

In this study we focus on large-scale vegetation dynamics in Europe (Fig 1) during the Last Interglacial (LIG, ~ 130,000–116,000 before present; all dates are given in calibrated years before present (hereafter abbreviated BP), where “present” is defined as 1950 CE) (Fig 1A) and the Early Holocene (~11,700–8000 BP, i.e., the period before the widespread adoption of agriculture in Europe) (Fig 1B). We start with a comparison of potential natural (i.e., climate-driven) (Figs 2A, 2B, 3A, 3B) and pollen-based (Figs 2C, 2D, 3C, 3D) vegetation reconstructions, revealing substantial differences between the two datasets. We then assess these differences by implementing an agent-based model (ABM) to track and quantify various impacts on interglacial vegetation, with a particular focus on vegetation burning by hunter-gatherers (Fig 4). It is important to emphasize that this study is primarily a modelling exercise based on currently available datasets from the broader body of research, which focuses on human-environment interactions at a continental scale [5,18–22].

Both study periods represent interglacial phases with broadly comparable vegetation dynamics [23]. The LIG has been proposed as a possible analogue for the Holocene and future environmental trends [24], hence the relevance of studying whether *Homo* played any role in the ecosystem dynamics of these times. In Europe, during both periods, humans subsisted as hunter-gatherers (foragers) who primarily relied on collection of wild resources [25] including plants, animals, and other natural resources. The absence of agriculture and domesticated animals during these periods may suggest that human impact on vegetation was minimal, with humans largely adapting to their natural environment rather than changing it. Ethnographic evidence [1–4] and a series of Early–Middle Holocene (~11,700–6000 BP) archaeological case studies [4,9–12,16,17] (Fig 1B) demonstrate that both past and recent hunter-gatherers used fire to alter vegetation for various purposes, including promoting useful plants, hunting, signaling, and clearing pathways [3,6,26]. Recently, evidence suggestive of such practices on a local scale has been published for the Neumark-Nord site in Germany, dating back to the LIG [27] (Fig 1A).

As a result of the inferred lower population sizes of foragers, researchers have characterized the LIG and the Early Holocene as periods with little to no human impact on landscapes compared to later phases. With fewer people interacting with the land, any ecological changes would have been relatively minor, particularly when compared to that of the larger agricultural populations with their different subsistence strategies. In addition, it is commonly assumed that human population size during the Mesolithic was larger than during the LIG [20,28]. As a result, only the activities of herbivores and/or natural fires are held responsible for transformations of natural vegetation cover during these periods, particularly during the LIG, and to have been mediated by climatic conditions [20,28–32].

While there may have been substantial differences in *Homo* population sizes between the Early Holocene and the LIG, such inferred differences have largely been assumed rather than directly observed. For example, there exist no solid archaeological data allowing a straightforward comparison between census (actual) populations of the LIG and the Early Holocene. Specifically, a direct comparison between the archaeological record of the Early Holocene and the LIG is unwarranted: these periods are separated by a full glacial cycle with considerable impact on site preservation and distribution patterns, and differ dramatically in the way sites can be identified as “Last Interglacial” or “Mesolithic”, creating a very strong bias against the number of LIG sites [33].

Demographic estimates usually rely on integrating multiple methods, scales, and proxies from archaeological sites [34], with genetic data playing an increasingly important role [35–38]. Solid data on Neanderthal population sizes during the LIG are not available. Although ancient DNA (aDNA) provides approximate effective population sizes–the number of reproductive individuals in an idealised population–for specific periods and regions occupied by Neanderthals [38–40]. A previous attempt to translate effective population sizes into census numbers yielded a broad estimate ranging from 5000 to 70,000 individuals, highlighting that these figures should be considered approximations rather than precise counts [41]. Notably, this estimate lacks specificity regarding particular regions or timeframes within the extensive span of Neanderthal existence.

Challenges remain for the Early Holocene since available local aDNA estimates do not provide continental-scale census human population sizes for the Mesolithic [38,42–46]. Other studies have used alternative methods and evidence to reconstruct Mesolithic demographic patterns within specific regions [34,47,48]. Continental-scale Early Holocene estimates relied on data and methods outside the scope of our research, including historical, ethnographic, and statistical modelling approaches [49–51]. It is possible that actual human populations were higher during certain periods [52]. Thus, comparing demographic patterns between the LIG and Early Holocene, and clearly relating them to hunter-gatherer impacts on landscapes, remains difficult.

The main research question addressed in this study is whether–and to which degree–hunter-gatherer activities could have impacted vegetation cover in Europe during the LIG and the Early Holocene. To address this question, we have set three primary objectives: 1) to evaluate the differences between potential natural vegetation (i.e., climate-based) as established via the CARAIB Dynamic Global Vegetation Model (DGVM) [53–55] and the reconstructed vegetation based on pollen obtained via the REVEALS model [28,56–58] for the selected time windows (Fig 4, step 1); 2) to generate potential scenarios of vegetation changes with outputs similar to REVEALS estimates due to megafauna plant consumption, anthropogenic and natural burning during the study periods (Fig 4, steps 2 and 3); and 3) to track, quantify and compare the calculated impact of Neanderthals and Mesolithic humans on vegetation for the most frequently generated scenarios (Fig 4, step 3).

To generate scenarios, we built upon a recently developed ABM called HUMan impact on LANDscapes (HUMLAND) [6,59,60], which was specifically adapted for the current study (Fig 4). ABMs provide opportunities to examine interactions within complex systems, especially when real-time experiments are not feasible. By simulating multiple interacting factors, ABMs generate potential scenarios of system behavior, which can then be compared to empirical data [61,62]. This approach has been already widely used to study past human–environment interactions [63–66]. HUMLAND was specifically designed to track and quantify different impacts on vegetation and to integrate various spatial datasets [5,19,21,22,28,58].

Building on insights gained from previous work [6], the current study focuses on two LIG time windows (mesocratic I and mesocratic II) and seven 500-year time windows during the Early Holocene, spanning 11,700–8200 BP. This allows, for the first time, the quantification of Neanderthal impact on interglacial vegetation and enables a comparison with the impact of Mesolithic populations. Additionally, for this study, we enhanced HUMLAND by adding hunting pressure on herbivores and refining the representation of their impact on vegetation during regeneration after disturbances. This major update provides a more realistic depiction of the role of megafauna and allows for greater precision in quantification while distinguishing different impacts on vegetation.

For HUMLAND 2.0 we needed an approach that would enable systematic and computationally efficient exploration of a wide range of scenarios represented by different combinations of parameter values within this ABM. We implemented a genetic algorithm, an optimization technique inspired by natural selection [67] for exploration of the parameter value space. Optimization involves testing various designs and adjusting model elements, such as agent behaviors and parameter values, to achieve a targeted outcome [68]. In our case, this outcome is a simulated vegetation cover that closely aligns with the past vegetation patterns (vegetation openness and distribution of dominant PFTs) represented by the REVEALS dataset. Genetic algorithms are widely recognized as a prominent approach for ABM optimization [69,70], though application in archaeological research has been relatively limited [71]. We present the first application of this algorithm to the HUMLAND ABM to identify combinations of parameter values that produce outputs similar to the REVEALS dataset. By using this innovative approach which integrates ABM, a genetic algorithm and various spatial datasets, we not only deepen our understanding of the history of human–environment interactions but also advance archaeological research by demonstrating the potential of genetic algorithms as an effective tool for optimizing complex multi-parameter models.

In this paper, our results are discussed in the context of broader questions about hunter-gatherer interactions with megafauna and demographic estimates for past populations, as detailed in the Discussion section. The study represents a methodical effort to explore potential scenarios that depict the dynamics of past interglacial ecosystems in Europe where we observe a discrepancy between modelled environments from climate simulations and those reconstructed via proxies.

## Materials and methods

Figure 4 provides an overview of our research steps. To achieve the first objective, CARAIB and REVEALS outputs were compared across all time windows. The CARAIB dataset represents theoretical potential natural vegetation (PNV) as shaped by climatic conditions (Figs 2A, B; Figs 3A, B; S1 and S3 Figs in S1 File.). This dataset is used as the starting point for every simulation run. The REVEALS dataset provides a reconstructed vegetation cover based on pollen data (Figs 2C, D; Figs 3C, D; S2 and S4 Figs in S1 File.), reflecting the result of the influence of various factors such as humans, megafauna, climate, and fires. In our ABM, the REVEALS data serves as a reference target vegetation cover for HUMLAND outputs.

CARAIB and REVEALS were compared for each time window in terms of two key aspects: the distribution of dominant PFTs and the vegetation openness across Europe [5,6,58]. While these two aspects are related, they do not constitute directly comparable model outputs. The first output indicates the dominant PFT: the primary vegetation type (trees, herbs, or shrubs) within a grid cell. Vegetation openness represents the percentage of vegetation density within grid cells. There is no direct correspondence between specific openness values and the PFT presence.

We used the previously developed HUMLAND ABM 1.0 [6,60] as the starting point for the major modifications needed to align this model with the scope of our current research. This led to the development and publication of the open-access HUMLAND 2.0 [59], which integrates new datasets relevant to our specific temporal focus, and has a more realistic representation of herbivory impact. As a result, HUMLAND 2.0 enables the study of *Homo*’s influence on herbivores via hunting and the subsequent effects on vegetation, including during regeneration phases. A crucial new aspect of this study is the combination of HUMLAND 2.0 with a genetic algorithm to systematically generate and analyze a range of potential scenarios.

The HUMLAND ABM was also designed to quantify the extent of different types of impacts on interglacial vegetation at a continental level. To meet the third objective, we selected parameter values with the highest frequency in the generated scenarios where outputs closely matched REVEALS. For these scenarios, we quantified the impacts of climate, megafauna, natural and human-induced fires. As a result, this study represents the first attempt to distinguish different sources of impact for the study periods. More specifically our study provides the first quantification of Neanderthal vegetation impact at a continental scale, allowing for direct comparison with that of later Mesolithic populations.

### HUMLAND ABM

In this study, we used as the base model the HUMLAND ABM 1.0 [6,60] implemented in NetLogo 6.2.2 [72]. This ABM explores vegetation dynamics, specifically PFT distribution and vegetation openness, in response to different factors, including climatic impact, human-induced and natural fires, and megafauna plant consumption. These factors are considered the most influential, widespread, and potentially observable at regional to sub-continental scales [4,6,20,28,30–32,73–75]. We made major changes to the base model and developed HUMLAND 2.0 [59]. We added megafauna impact on vegetation regeneration (as detailed below). This included the introduction of hunting pressure, allowing for the exploration and quantification of the potential effects of Neanderthals and Mesolithic humans on herbivore populations.

HUMLAND 2.0 operates at a temporal resolution of one year and a spatial resolution of 10 km × 10 km, with each simulation running for a maximum of 1000 steps. We selected this spatial resolution as a compromise between the varying input data resolutions ranging from 1 km × 1 km to 100 km × 100 km, the localized yet varied scale of hunter-gatherer vegetation burning (estimated based on ethnographic evidence to range from several kilometers to 100 km^2^), and the continental scope of the model [3,4,6]. A larger grid size could obscure the localized effects of foragers by blending them with other factors such as climatic changes. The number of steps (1000) was chosen to ensure that each simulation reaches an equilibrium state–where the key observations stabilize and do not substantially vary–usually occurring around step 450 [6]. For further analysis, primary HUMLAND output (mean vegetation openness and the mean number of grid cells dominated by herbs and trees) were recorded after step 450, when equilibrium is reliably reached. These outputs are collected only for grid cells that have both CARAIB and REVEALS values.

HUMLAND 2.0 is run separately for two discrete LIG time windows representing the period of maximum forest distribution in Europe and for four discrete Early Holocene 500-year time windows, spanning 10,200–8200 BP. Each simulation run is independent and does not overlap with others. The chosen time windows align with the temporal resolution of the datasets provided by REVEALS. The period between 11,200 and 10,200 BP was included in the CARAIB–REVEALS comparison but excluded from the simulations and the generation of potential scenarios via the genetic algorithm due to the difficulty of distinguishing human-induced changes from climatic changes during the glacial–interglacial transition at the onset of the Holocene [76,77].

Here, we provide a brief introduction to HUMLAND 2.0. Further details can be found in Nikulina et al. [6] and in the Overview, Design concepts and Details (ODD) document for HUMLAND 2.0 [59].

Each simulation step starts with a climatic impact affecting vegetation regrowth after fires or consumption by megafauna (Fig 4). Since average recovery times (the number of years for vegetation to fully recover in accordance with a PNV PFT) were not available for the four PFT categories, we used estimates from the CARAIB model: herbs recover in seven years, needleleaf trees and shrubs in 43 years, and broadleaf trees in 30 years [6]. These specified recovery periods refer to the point at which a PFT becomes the first dominant PFT following a disturbance. Generally, vegetation recovery depends on different factors including weather conditions, animal activity, season of disturbance, and even presence of specific nurse plants [78–80]. Various case studies report recovery times for vegetation cover ranging from several months to several years, depending on specific conditions; the recovery of plant community structure (e.g., species richness and dominance patterns) may take several decades [75,81–84]. In some cases, full ecosystem recovery can take more than seven years [85,86].

These aspects to a certain degree are reflected in HUMLAND. When vegetation recovery begins following fire or vegetation consumption, vegetation openness decreases. This indicates that some vegetation cover reappears in HUMLAND within one year (one simulation step) after disturbance. In the following steps, vegetation progressively regains density until it reaches the PNV openness in accordance with the CARAIB data. This recovery process may be delayed if additional disturbances occur during the regeneration phase. The vegetation openness recovery rate is calculated by taking the difference between current vegetation openness (after disturbance) and the PNV openness, then dividing this difference by the average recovery time. During each simulation step, this recovery rate is subtracted from the current openness until it reaches the PNV openness.

PFT recovery follows a straightforward process in HUMLAND. Based on the CARAIB estimates mentioned above, bare ground is replaced by herbs after seven simulation steps. Afterwards, herbs may be replaced by trees or shrubs after required number of steps, depending on the PNV PFT estimated by CARAIB.

HUMLAND 2.0 has adjustable parameter values for simulation runs (Table 1). The minimum and maximum values for most of these parameters were established previously [6]. HUMLAND includes several switches that allow for different combinations of impacts on vegetation, enabling their addition or removal as needed.

**Table 1:** HUMLAND 2.0 parameter overview.

Parameters	Associated source of impact	Units/Type	Values		Description
			Min	Max	
Territory_impacted_by_thunderstorms	Natural fires	%	0	100	Percentage of terrestrial grid cells impacted by thunderstorms per simulation step.
Natural_fires		Boolean	True/False		Indicates the presence or absence of thunderstorms during one simulation run.
Hunting_pressure	Hunter-gatherers, megafauna plant consumption	%	0	100	Reduces the estimated maximum potential megafauna plant consumption.
Megafauna_impact	Megafauna plant consumption	Boolean	True/False		Indicates the presence or absence of megafauna plant consumption during one simulation run.
Humans	Hunter-gatherers	Boolean	True/False		Indicates the presence or absence of anthropogenic impact during one simulation run.
Number_of_groups		Groups	0	4000	Specifies the number of human groups present in the study area during one simulation run.
Accessible_radius		Grid cells	0	5	Defines the territorial range within which humans move and set fires around their campsites.
Openness_criteria_to_burn		%	9	100	Specifies the threshold openness value below which humans set fires in grid cells dominated by trees or shrubs.
Movement_frequency_of_campsites		Steps	0	1000	Defines the frequency of campsite relocation by specifying the number of simulation steps after which relocation occurs.
Campsites_to_move		%	0	100	Specifies the percentage of campsites relocated at a given simulation step.

Natural ignition from thunderstorms is determined by the probability of ignition, which depends on the time elapsed since the last burning episode and the natural fire return intervals of the specific PNV PFT in that grid cell. Thus, the model accounts for the variations in the dominant PFT and probability of ignition and spread is different for needleleaf trees, broadleaf trees, shrubs and herbs. Fire return intervals were obtained via so-called “space-for-time” substitution, based on remote sensing data of fire activity [6,87].

Due to the continental scope of our study, we assumed that all fires replace the vegetation of a grid cell with bare ground in HUMLAND. However, observations from different regions indicate that fires do not always result in total vegetation loss; their impacts can range from minor fire scars to complete change of vegetation cover [79]. Predicting the exact consequences of fires on plant communities is challenging due to variations in fire size, frequency, and intensity [78,88]. While our assumption simplifies the modelling process, it may introduce some uncertainty into our results.

After anthropogenic and natural burning events, fires can spread to any of the eight neighboring grid cells (Moore neighborhood) based on their probability of ignition which depends on the PNV PFT. Fires cannot occur and spread on water bodies, bare ground and high mountains.

To more accurately depict the effects of megafauna on vegetation in HUMLAND 2.0 during the regeneration phase, and to explore scenarios where vegetation dynamics are not driven by anthropogenic fires, we implemented two key modifications in the initial model version: a reduction in the intensity of animal impact due to hunting pressure and due to the state of vegetation openness at the time of consumption.

Humans are often mentioned as being responsible for the Quaternary megafauna extinction and further decline of functional diversity [19,89–93]. In addition, the localized disruptions in herbivore populations preceded the widespread megafauna extinction, given the shared preferences for game species between Neanderthals and early modern humans in Eurasia [94–98]. Given this, we introduced the “Hunting_pressure” parameter (Table 1), which reduces the estimated potential maximum plant consumption (as described in the Datasets used in the HUMLAND ABM section). This parameter affects megafauna plant consumption even when hunter-gatherers do not burn vegetation. In our model, this parameter does not impact LIG megafauna plant consumption on the British Isles because humans were not present or had sparse occupation there during this time [99].

Besides hunting, the intensity of megafauna impact is determined by the state of vegetation openness. Many herbivores prefer areas with secondary vegetation and relatively open regrowth zones following disturbances such as fire [100–104] because it increases the nutrition and palatability of new plants [105]. Consequently, fire attracts herbivores, which, in a reciprocal relationship, impact vegetation regeneration and fire behaviour [75]. Thus, areas with greater openness tend to experience more substantial herbivore impact. This serves as the second determinant of megafauna impact intensity within HUMLAND 2.0. Due to these two key modifications in megafauna plant consumption, animals now interact with grid cells at every simulation step, including those that are regenerating after fires.

Following the constraints imposed by hunting pressure, the resultant value of megafauna plant consumption of a grid cell after hunting (V_h_) is further limited by the current vegetation openness (O_i_) of the grid cell. This restriction yields the final estimate of megafauna NPP (Net primary productivity) metabolization (V_m_) through formula 1:


$$V_{m}=\frac{O_{i}}{100}\times V_{h}$$ (1)

Afterwards, the V_c_ value quantifies the percentage of vegetation consumed in each grid cell, excluding water bodies and high mountains, using formula 2:


$$V_{c}=\frac{V_{m}}{V_{n}}\times 100$$ (2)

V_n_ represents the current NPP of the consumed grid cell. The resulting V_c_ value is then combined with vegetation openness to reflect the impact of megafauna. In HUMLAND, megafauna can only consume vegetation in grid cells that are not completely open (vegetation openness is less than 100%). After the megafauna plant consumption of a grid cell, the current NPP of this grid cell is reduced based on the calculated percentage of consumed vegetation (V_c_).

In the beginning of each simulation run with human-induced fires, forager campsites are distributed randomly. During the LIG runs Neanderthals do not occupy or burn vegetation in the British Isles [99], whereas Mesolithic hunter-gatherers are present in this region.

Regarding human-induced vegetation burning, three parameters influence its intensity as demonstrated by the sensitivity analysis of HUMLAND [6]: “Number_of_hunter-gatherer_groups”, “Accessible_radius”, and “Openness_criteria_to_burn”. Ethnographic evidence shows that hunter-gatherers burn vegetation for various reasons across different vegetation types [3,106]. The “Openness_criteria_to_burn” parameter partially reflects this variability. Higher values of this parameter result in more frequent burning by hunter-gatherers, targeting both relatively closed and open landscapes. In some cases, these landscapes may not have fully regenerated to their original vegetation openness level after previous disturbances such as fires or consumption. As a result, hunter-gatherers do not exclusively burn climax vegetation but may also target areas that have not fully recovered yet.

HUMLAND can store the last agent responsible for vegetation changes in grid cells at each simulation step. It is tracked through two grid cell variables: “last_agent_impacted_pft” and “last_agent_impacted_openness”. Updating the “last_agent_impacted_pft” variable requires an agent to replace the current dominant PFT with bare ground. This can occur through natural or anthropogenic fires, as every burning episode in HUMLAND results in vegetation being replaced by bare ground. Additionally, climate-induced changes can modify this parameter during the regeneration phase. It is important to note that megafauna can only update the “last_agent_impacted_pft” parameter when their impact is strong enough to transform vegetation by replacing a dominant PFT.

The “last_agent_impacted_openness” variable is updated when an agent induces a substantial transformation in the vegetation openness of a grid cell. This transformation is guaranteed in the case of a fire event, as it sets the vegetation openness of the burnt grid cell to 100% (bare ground). If, during vegetation regrowth, the vegetation openness of a grid cell closely aligns with CARAIB estimates (i.e., the difference between CARAIB and HUMLAND openness values is equal to or less than 10%), then “last_agent_impacted_openness” is modified due to climatic influence.

Given the relatively low-intensity impact of megafauna on all grid cells (i.e., V_c_ is below 1% per simulation step for most of grid cells), we assumed that for megafauna to be recognized as an agent responsible for changing vegetation openness of a grid cell, animals must effect a transformation to some extent comparable to that induced by fires and climate. Thus, if the vegetation openness of a grid cell deviates by more than 10% from CARAIB’s openness estimates as a result of continuous and sustained megafauna impact over 10 simulation steps (equivalent to 10 years in HUMLAND), and in the absence of influence from other agents, megafauna can be identified as the agent responsible for the transformation in vegetation openness for that specific grid cell.

### Datasets used in the HUMLAND ABM

We used the Spatial Analyst and Data Management toolboxes in ArcMap 10.6.1 to standardize the spatial extent and resolution (10 km × 10 km) of the datasets used in this study (S2 Table in S1 File). The datasets, along with their original grid cell sizes, are listed below. Each newly generated 10 km × 10 km grid cell was assigned values from larger grid cells in the original datasets. Additionally, certain datasets were reclassified as detailed below. For this study, we incorporated input datasets covering two LIG time windows, corresponding to the period of maximum biomass development in Europe, and seven Early Holocene time windows.

To ensure consistency in our analysis, we excluded Anatolia, Cyprus, and the Balkans from all time windows considered in this study (Fig 1). These regions have the earliest evidence of agriculture in Europe [107,108]. By excluding them, we can focus on the impact of hunter-gatherer vegetation burning while minimizing potential factors related to agricultural activities during the Holocene.

The initial landscape is reconstructed via the DEM Global Topography 30 Arc-Second (~1 km) elevation dataset (GTOPO30) (www.usgs.gov) [109,110] Water Information System for Europe (WISE) (https://water.europa.eu/) and CARAIB outputs which are used as a starting point for all simulation runs [55,111–113]. Details on the CARAIB model setup can be found in Supporting Information.

CARAIB outputs used in this ABM include distribution of fractions of 26 PFTs (PNV distribution), PNV vegetation openness, and potential natural NPP per 26 km × 26 km grid cell [5,53]. CARAIB simulations are based on climate simulations performed with the iLOVECLIM climate model. It includes the VECODE reduced-form vegetation model [114], which computes plant and soil behaviours necessary for simulating first-order vegetation-climate feedback in climate models [5]. In turn, CARAIB is a more comprehensive mechanistic vegetation model that simulates vegetation dynamics based on interactions with climatic and soil conditions. It also models heterotrophic respiration and litter/soil carbon dynamics [55].

To simulate Holocene climate evolution, we applied iLOVECLIM in a transient run (where the climate model runs continuously over a specified period). The outputs were resampled (averaged over the years) to match 500-year-long REVEALS time windows, ensuring alignment between CARAIB and REVEALS datasets for comparative analysis.

In contrast to the Holocene, aligning CARAIB and REVEALS outputs is challenging for the LIG. This difficulty arises from the fact that this stage was identified based on pollen assemblages, and the timing and duration of the LIG varied across different regions in Europe [23,115]. As a result, the exact start and end points of this period remain unclear. In our research, precisely aligning REVEALS time windows with corresponding CARAIB outputs is critical. While achieving a perfect match may not currently be possible for the LIG, we have chosen to focus on the REVEALS mesocratic I (*Quercus* zone) and II (*Carpinus* zone) time windows corresponding to the maximum biomass development [116,117].

To select CARAIB output for the time slice with maximum forest fraction during the LIG, we conducted a series of transient climate simulations [22], followed by cross-validation through equilibrium simulations (climate model is run under fixed forcing conditions until it reaches a state of equilibrium) for three specific time slices characterized by high forest fractions in the transient runs: 120,000 years BP, 124,000 years BP, and 128,000 years BP. Our tests (not shown) determined that 128,000 years BP represents the peak of forest fraction during the LIG within our modelling setup. The corresponding CARAIB output was used as the starting point for two LIG time windows during LIG HUMLAND 2.0 runs. While we acknowledge that using this LIG CARAIB output may contribute to discrepancies between this dataset and REVEALS estimates, and that this can be considered a limitation of our study, it currently remains the only viable approach for running HUMLAND simulations for the LIG.

Before running HUMLAND simulations, CARAIB outputs were transformed and compared against pollen-based estimates of plant cover initially reconstructed for 1° × 1° (~100 km × 100 km) grid cells for each time window. These estimates were obtained from the REVEALS model which is based on pollen records from multiple-sized lakes and bogs and/or large lakes (>50–100 ha) [28,56–58]. The REVEALS dataset also serves as the optimization target for genetic algorithm experiments. We compared CARAIB and REVEALS following the approach used in HUMLAND [6]. Both CARAIB and REVEALS PFTs were included in the current simulations and analyzed within four PFT categories: needleleaf and broadleaf trees, shrubs and herbs (Fig 3). The corresponding table between CARAIB PFTs and REVEALS plant taxa and morphological types is available in Supporting Information (S1 Table in S1 File). It is important to note that the PFTs used in this study were designed for continental-scale dataset comparisons, leading to merging certain categories, such as dwarf shrubs and shrubs.

The results from REVEALS are influenced by several input parameters, including original pollen counts, relative pollen productivity (RPPs) and their standard deviations, fall speed of pollen, basin type (lake or bog), size (radius, m), maximum extent of the regional vegetation (km), wind speed (m.s^−1^), and atmospheric conditions [58]. For our study, we used REVEALS reconstructions for the Holocene, based on 31 plant taxa [58], and for the LIG, based on 30 plant taxa [28]. Some taxa from the original pollen diagrams are absent from our pollen-based reconstructions, as pollen productivity estimates are not available. While pollen productivity estimates are available for many taxa, previous studies have stressed the importance of minimizing the inclusion of strict entomophilous taxa in REVEALS reconstructions to improve accuracy [58,118]. As a result, some categories may be over- or underestimated depending on the taxa available within each category. In our study, we used REVEALS reconstructions for the LIG and the Early Holocene based on the work of Pearce et al. and Serge et al., with details on the applied protocols available in the respective studies [20,28,58].

The REVEALS model estimates vegetation cover based on pollen data but does not account for the presence of bare soil. To address this limitation, some studies have improved land-cover reconstructions by incorporating bare ground fractions derived from dynamic vegetation model outputs such as the Lund–Potsdam–Jena General Ecosystem Simulator (LPJ-GUESS), or by considering the spatial extent of glaciers [119,120].

Besides dominant PFTs, we used potential natural (CARAIB) and pollen-based (REVEALS) vegetation openness in percentages (Fig 2). REVEALS estimates for vegetation openness include the percentage of all herbs and *Calluna vulgaris* for each grid cell [6,58,121]. In contrast to REVEALS, CARAIB estimates vegetation openness for two vertical levels: lower (herbs, shrubs and bare ground) and upper (trees). We classified bare ground and herbs as indicators of open areas, while trees and shrubs were classified as closed areas. For each vertical CARAIB level, the maximum possible openness value is 100%, representing the percentage of an area not covered by shrubs or trees. Consequently, the highest combined openness value for a grid cell is 200%, indicating a completely open area containing only bare ground and/or herbs. To align CARAIB with REVEALS in terms of vegetation openness, we assigned a single openness value per grid cell in the CARAIB dataset, using the smaller value between the two levels to represent the fraction of the area without trees or shrubs. By applying this transformation, both REVEALS and CARAIB datasets were adjusted to represent comparable distributions of dominant PFTs and vegetation openness.

We combined CARAIB NPP with potential maximal megafauna plant consumption (i.e., metabolization of NPP by wild terrestrial mammals ≥ 10 kg) to estimate the percentage of vegetation consumed by megafauna (see section HUMLAND ABM). Since body mass is a key functional trait influencing animal impact, we adopted the 10 kg threshold, a widely used benchmark in ecological studies [19,90,122,123]. The potential maximal vegetation consumption of wild herbivore communities was first calculated across the continent prior to the extensive influence of humans on landscapes in the form of consumed kg/km^2^ per year per 30 km × 30 km grid cell [19]. We used the obtained dataset for the LIG runs as the maximal possible megafauna plant consumption during this time. From this dataset we excluded the species absent from the Holocene fossil record, including straight-tusked elephants (*Palaeoloxodon antiquus)* [122,124,125]. As a result, the obtained dataset reflects maximal possible megafauna plant consumption during the Early Holocene because it considers all areas of the continent that could have been frequented by the species based on climatic suitability, when the actual range of these species had been already substantially reduced due to human impact in the Late Pleistocene [19,122]. Given the absence or sparse presence of Neanderthals in the British Isles during the LIG [99], we added an additional spatial layer to HUMLAND 2.0. This layer defines areas with no hunter-gatherer impact on megafauna plant consumption, and where hunter-gatherers were absent in the LIG ABM runs.

To incorporate LIG sea level differences in HUMLAND, we used available reconstructions and estimates of past sea levels. Specifically, for Northwest Europe, we utilized coastline reconstructions based on the work of Cohen et al. [126]. However, similarly detailed reconstructions were unavailable for other European regions. Consequently, we applied a uniform sea level rise of 6 m for the remainder of Europe during the LIG. This value is derived from global high-stand estimates, which indicate multiple peaks ranging from 2–3 m to 5.5–9 m a.s.l. [127,128]. With these considerations, we defined the study area for the LIG datasets by excluding regions falling within the reconstructed North European LIG sea levels and currently situated below 6 m a.s.l. (Fig 1A). Because no comprehensive reconstructions exist for the distribution of major rivers and lakes in Europe during the LIG, we adopted their modern distributions based on the WISE dataset.

In HUMLAND, areas with closed vegetation can only transition to more open vegetation after fires or plant consumption. Our ABM can only create a match with REVEALS estimates if the initial CARAIB vegetation openness (climax vegetation) is equal to or less than pollen-based estimates (i.e., more closed vegetation can open further) or where shrubs or trees can transition to bare ground and herbs. Consequently, all grid cells that did not meet these criteria were excluded from the CARAIB–REVEALS comparison and from the genetic algorithm experiments.

### Genetic algorithm

We used the genetic algorithm optimization technique to generate potential scenarios and determine the parameter values for HUMLAND 2.0 that are needed to produce ABM outputs closely aligned with the REVEALS data (Fig 4). This technique was originally developed in the 1960s–1970s by John Holland and his collaborators [129,130]. A genetic algorithm encodes an objective function as arrays of bits or character strings, representing chromosomes, and employs genetic operators to manipulate these strings. Solutions are selected based on fitness, enabling the algorithm to converge toward an optimal solution to a problem in hand [130]. This process involves the following steps: 1) encoding solutions into strings; 2) defining a fitness function and selection criterion; 3) creating a population of individuals and evaluating their fitness; 4) evolving the population by generating new solutions through crossover, mutation, and fitness-proportionate reproduction; 5) selecting new solutions based on their fitness and replacing the old population with better individuals; and 6) decoding the results into the solution(s) to the problem [130].

We implemented the genetic algorithm and subsequent analysis of the modelling results using R (RStudio Version 1.3.1093, [131]). We used the *nlrx* package which explores various model parameters within predefined ranges to minimize a fitness criterion [132]. Our optimization goal was to minimize two differences: 1) the discrepancy between mean vegetation openness obtained from REVEALS (O_r_) and HUMLAND (O_h_), and 2) the difference in the mean percentage of grid cells dominated by trees from REVEALS (T_r_) and HUMLAND (T_h_). Thus, we used the two fitness functions (formulas 3 and 4):


$$f(O)=\frac{\left|O_{r\;}-O_{h\;}\right|}{100}\;$$ (3)

and


$$f(T)=\frac{\left|T_{t}\;-\;T_{h}\right|}{100}$$ (4)

O is mean vegetation openness, and T is the mean percentage of grid cells dominated by trees. These values were calculated only for grid cells that contained both REVEALS and CARAIB estimates. As a result, we conducted two main groups of genetic algorithm experiments. The first group focused on minimizing the difference in mean vegetation openness obtained via REVEALS and HUMLAND. The second group aimed to minimize the REVEALS–HUMLAND difference in the percentages of grid cells dominated by trees. For each fitness function per time window, we conducted 60 separate genetic algorithm experiments using different random seeds for the following three subsets of experiments: 1) megafauna impact; 2) megafauna impact and natural fires; 3) megafauna, natural and human-induced fires. All experiments include hunting pressure by foragers and vegetation regeneration via climatic impact. Consequently, we obtained a total of 360 genetic algorithm results per time window, and 2160 results in total for all time windows.

As we had already identified the most influential parameters for human-induced vegetation changes and their minimum and maximum values in HUMLAND [6] (Table 1), we used these values only for those specific parameters (Table 2). In the genetic algorithm experiments we also incorporated the “Hunting_pressure” parameter which is estimated as a percentage ranging from 0% to 100%. The “Territory_impacted_by_thunderstorms” had a constant 0.04% value in accordance with the decadal lightning observations for Europe [133]. For this parameter we used modern estimates due to the absence of continental LIG and Early Holocene thunderstorm frequency values.

**Table 2:** Genetic Algorithm setup details. A black dot indicates that a variable was optimized within its specified minimum and maximum values (as outlined in Table 1), whereas a white dot signifies that the variable remained constant. The experiment subsets are categorized as follows: 1) megafauna impact; 2) megafauna impact combined with natural fires; and 3) megafauna impact, natural fires, and human-induced fires.

Parameter	Experiment subset 1	Experiment subset 2	Experiment subset 3
Territory_impacted_by_thunderstorms	0.04	0.04	0.04
Megafauna_impact	True	True	True
Natural_fires	False	True	True
Humans	False	False	True
Number_of_hunter-gatherer_groups	○	○	●
Accessible_radius	○	○	●
Openness_criteria_to_burn	○	○	●
Hunting_pressure	●	●	●
Campsites_to_move	0	0	0
Movement_frequency_of_campsites	0	0	0

The genetic algorithm was configured with a population size (popSize) of 30 and a total of 20 iterations (iters). The fitness function output measurements were recorded after step 450 when HUMLAND reaches its equilibrium [6].

To assess the effectiveness of the genetic algorithm results, we first calculated the percentage of HUMLAND scenarios that produced outputs comparable to REVEALS estimates. Specifically, we determined the proportion of scenarios where 1) the mean vegetation openness differs from REVEALS by 10% or less, and 2) the percentage of grid cells dominated by trees differs from REVEALS by 10% or less. This calculation provided a quantitative measure of the overall success of each experimental subset.

Afterwards, for the successful scenarios, we computed Pearson correlation coefficients (PCC). These correlations were then visualized as a correlation matrix using the *corrr* and *ggcorrplot* packages [134,135]. Additionally, we performed principal component analysis (PCA) utilizing the *FactoMineR* package [136]. To explore the parameter values for generated scenarios similar to REVEALS and to identify the most frequently occurring value ranges, we used box and violin plots created via the *ggplot* package [137] and measures from descriptive statistics (mean, standard deviation and mode).

To evaluate the visibility of each agent’s impact on vegetation at the continental level, we calculated the mode (the most frequent value in a data set) for the scenarios that led to the similar output with REVEALS. We calculated the mode values for each generated parameter value distributions separately within each time window. Subsequently, we selected combinations of the generated parameter values that closely matched these separate mode values. In cases where parameter value distributions had several modes, we selected multiple combinations. Using the selected parameter combinations, we conducted additional HUMLAND simulation runs (S6 Table in S1 File). Throughout these runs, HUMLAND tracked for each grid cell (excluding water bodies and high mountains) the last agent that influenced the vegetation openness of the grid cell and modified the first dominant PFT of that grid cell. The obtained observations were averaged and presented in bar charts for LIG and the Early Holocene separately.

## Results

### Comparison of REVEALS and CARAIB datasets

**Fig 5:**
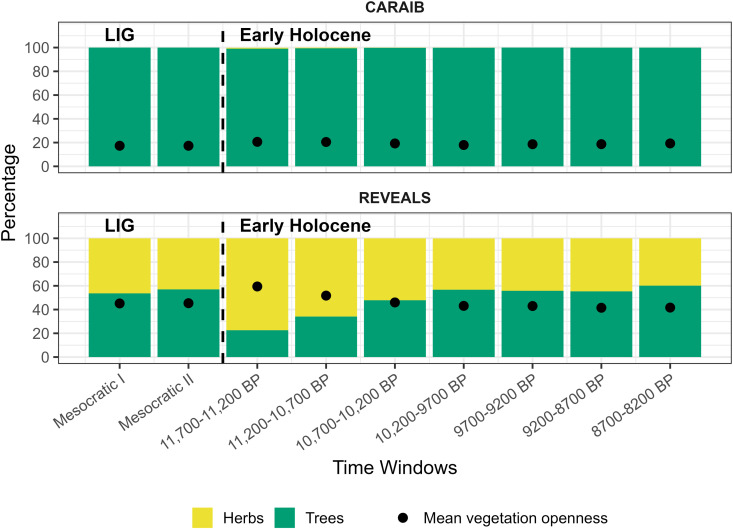
CARAIB–REVEALS comparison of mean vegetation openness (black dots) and the mean percentage of grid cells dominated by herbs (yellow) and trees (green) for the LIG and the Early Holocene.

The results of the CARAIB–REVEALS comparison for all time windows are shown in Fig 5. The comparative outcomes for the two LIG time windows are derived from a notably smaller set of 10 km × 10 km grid cells (1211 and 1277) than for the Early Holocene, where a substantially larger number of grid cells was considered in our study, ranging between 14,703 and 16,478 depending on the specific time window. The REVEALS grid cells included in the analysis are shown in Figs 2C, 2D, 3C, and 3D for two specific time windows. The other time windows are presented in S2 and S4 Figs in S1 File.

Across all time windows CARAIB consistently exhibits substantially higher mean percentages of grid cells dominated by trees compared to REVEALS (Fig 5, shown in green). Additionally, a consistent trend is observed in mean vegetation openness estimates, with CARAIB showing substantially lower values than REVEALS (Fig 5, shown by dots). The mean percentage of grid cells dominated by herbs follows a similar pattern (Fig 5, shown in yellow). Thus, pollen-based reconstructions indicate a more open environment than CARAIB.

Intriguingly, our results reveal a noteworthy inversion in the mean percentage of grid cells with herbs and trees in the REVEALS estimates (Fig 5, bottom figure) between 10,700–9700 BP. In the initial phases of the Early Holocene (11,700–10,200 BP), REVEALS reconstructions show that herb-dominated grid cells outnumbered those dominated by trees. However, from 10,200–8200 BP, there is a shift toward the predominance of tree-dominated grid cells. This pattern remains relatively stable, with a slight increase occurring at 8700–8200 BP. The LIG time windows show a comparable pattern, with notably similar variations in the proportions of grid cells dominated by herbaceous and arboreal vegetation. Based on the results of this CARAIB–REVEALS comparison we selected the time windows for HUMLAND runs: two LIG and four Early Holocene (10,200–8200 BP) time windows (Fig 5).

### Vegetation dynamics without human-induced burning: megafauna plant consumption, hunting, and natural fires

There are two experimental subsets that excluded human-induced fires: 1) megafauna impact, where fires were completely absent, and 2) megafauna impact with natural fires (Table 2). In both subsets, animal hunting was present, meaning the potential maximum megafauna plant consumption was reduced according to the values specified by “Hunting_pressure”.

The instances where ABM results align with the REVEALS estimates, particularly concerning the PFT distribution, are rare (Table 3). Thus, our results show that it is almost impossible to produce scenarios similar to the pollen estimates without fires and specifically without burning by foragers.

**Table 3:** Percentage of possible scenarios with output similar to REVEALS without anthropogenic fires. In these scenarios humans do not engage in vegetation burning, but they exert hunting pressure on herbivores.

Time windows	No fire events		Natural fires only	
	PFT distribution	Mean vegetation openness	PFT distribution	Mean vegetation openness
Mesocratic I	0%	66%	0%	65%
Mesocratic II	0%	69%	23%	71%
10,200–9700 BP	0%	0%	0%	63%
9700–9200 BP	0%	0%	0%	82%
9200–8700 BP	0%	0%	0%	90%
8700–8200 BP	0%	0%	0%	100%

**Fig 6:**
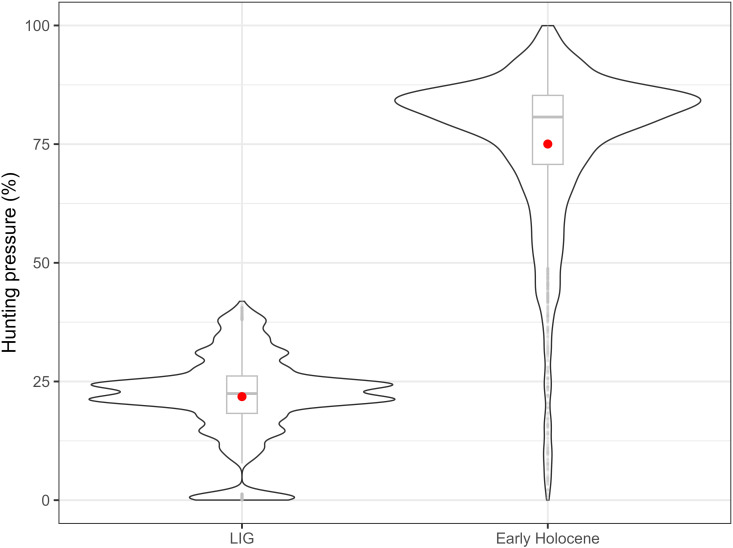
Summary statistics and values’ distribution of the “Hunting_pressure” parameter values required to generate HUMLAND scenarios with output similar to REVEALS without anthropogenic fires. Humans do not engage in vegetation burning, but they exert hunting pressure on herbivores. The dot indicates the mean value for each dataset. For the LIG, most simulations matching REVEALS outputs have “Hunting_pressure” values around 20–25%, whereas for the Early Holocene, they typically cluster around 80–90%.

In HUMLAND scenarios without anthropogenic fires but producing vegetation openness outputs consistent with the REVEALS data, humans would have needed to reduce megafauna pressure through hunting. During the LIG, this would require decreasing megafauna plant consumption by 20–25% to match the openness levels shown in the REVEALS estimates (Fig 6). During the Early Holocene, achieving the openness levels shown by REVEALS data would require a much greater impact on megafauna, with 80–90% of the animal population removed via hunting (Fig 6). In other words, without hunting, megafauna impact would have resulted in landscapes different than those reconstructed by REVEALS.

### Vegetation dynamics with human-induced burning: megafauna plant consumption, hunting, natural and anthropogenic fires

Human-induced burning is incorporated into the third experimental subset, alongside natural fires and megafauna impact (Table 2). HUMLAND parameters were adjusted using a genetic algorithm within their predefined ranges (Table 1) to generate outputs closely matching REVEALS data. As a result, the majority of generated scenarios had results that matched REVEALS estimates (Table 4). Further analyses, including PCA (S3 and S4 Tables in S1 File) and PCC (S5 Fig in S1 File.), were performed only on scenarios closely matching the REVEALS data.

**Table 4:** Percentage of possible scenarios with output similar to REVEALS with anthropogenic fires. These scenarios include the combined direct impact of all agents on vegetation: human induced and natural fires, and megafauna plant consumption.

Time windows	PFT distribution	Mean vegetation openness
Mesocratic I	89%	98%
Mesocratic II	94%	99%
10,200–9700 BP	98%	100%
9700–9200 BP	98%	100%
9200–8700 BP	98%	100%
8700–8200 BP	98%	100%

PCC showed that the variables within the LIG dataset have both positive (i.e., when one increases, the other also increases) and negative correlations, while in the Early Holocene results, correlations are exclusively negative (i.e., an increase in one factor coincides with a decrease in another) (Fig S5 in S1 File). The magnitudes of the correlation coefficients between parameters are generally absent, low or modest for both periods. PCA results show that contribution of some variables to principal components (i.e., new variables that are derived from an original set of variables to reduce the dimensionality of data) varies over time and across genetic algorithm experiment groups (S3 and S4 Tables in S1 File). Consequently, it is difficult to identify a single parameter or specific combination of parameters that consistently has the greatest influence on model outputs. A distinct result is that the absolute loadings (i.e., how much a variable contributes to the component) of the “Hunting_pressure” parameter are overall lower for LIG results compared to the Holocene runs.

**Fig 7:**
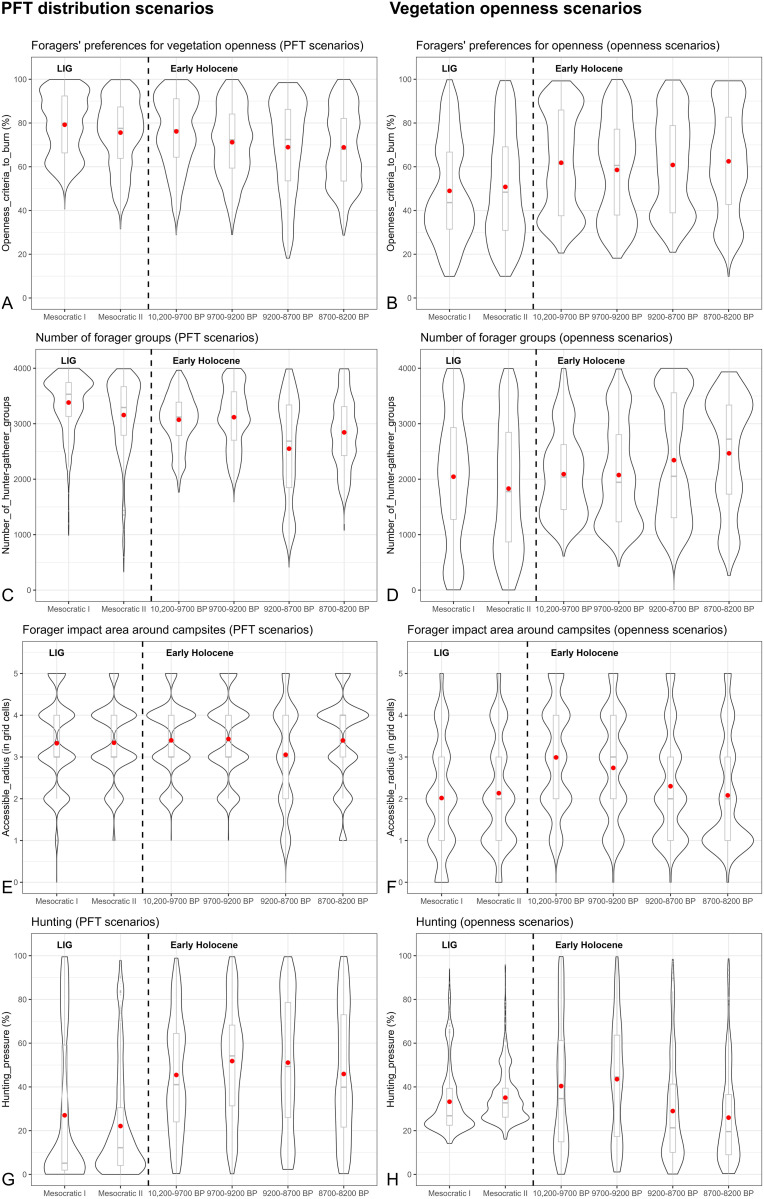
Summary statistics and distribution of the parameter values required to generate scenarios with output similar to REVEALS for PFT distribution (A, C, E, G) and vegetation openness (B, D, F, H) with hunting and anthropogenic fires. The dot indicates the mean value for each dataset.

The range of parameter values required to produce scenarios comparable to REVEALS outputs varies across time periods and experiments (Fig 7). A consistent observation is that higher values for the “Openness_criteria_to_burn” are necessary to produce PFT distribution scenarios (with means of 77% for the LIG and 71% for the Early Holocene) compared to vegetation openness scenarios (with means of 49% for the LIG and 60% for the Early Holocene) (Figs 7A, B). A similar trend is noted for the “Number_of_groups” parameter (Figs 7C, D), where the mean values for tree distribution scenarios are 3266 for the LIG and 2895 for the Early Holocene, while for vegetation openness scenarios, the means are 1936 for the LIG and 2243 for the Mesolithic. Overall, within each group of genetic algorithm experiments, the values of these parameters for the Neanderthal and Mesolithic periods are similar, showing minimal differences between the LIG and Early Holocene ranges.

The accessible radius values for the PFT scenarios are consistent, with a mean around three and the most frequent values at three and four grid cells around campsites across most time windows (Fig 7E). In the vegetation openness scenarios, the Neanderthal mean radius is around two. However, the area impacted by Mesolithic humans shows a reduction from three grid cells during 10,200–9700 BP to an average of two grid cells between 8700–8200 BP, with most values at one during this time window (Fig 7F).

The results indicate significant variability in potential hunting pressure across different study periods within the PFT scenarios: an average decrease of 24% in megafauna plant consumption is needed during the LIG, compared to 48% during the Early Holocene (Fig 7G). Conversely, the vegetation openness scenarios show similar average hunting pressures for both time periods, around 34% (Fig 7H). However, the most frequent values differ between the periods. For the LIG, vegetation openness scenarios typically require a reduction in plant consumption by megafauna ranging from 21% to 39%, whereas for the Early Holocene, the range is much broader, from 1% to 82%. The PFT scenarios generally indicate hunting pressure of 0% to 4% for the LIG, and 0% to 67% for the Mesolithic. Similarly, the vegetation openness scenarios reveal that the most common values for the “Openness_criteria_to_burn” vary between periods: ranging from 23% to 48% for the LIG and from 36% to 69% for the Early Holocene (Fig 7B). For the PFT scenarios, the most common values for this parameter remain relatively close across the periods (Fig 7A).

### Continental scale visibility of different types of impact

**Fig 8:**
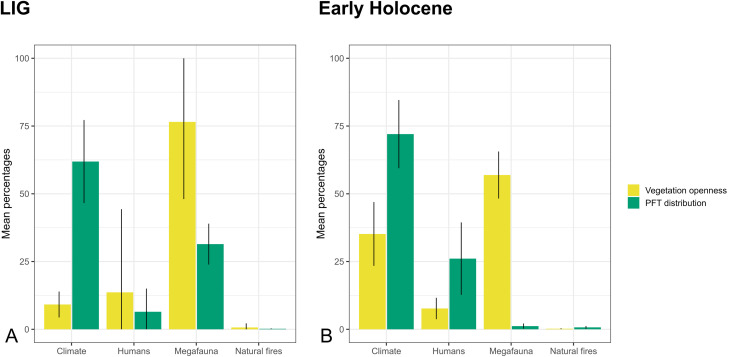
Mean percentages of grid cells modified by different agents during the HUMLAND equilibrium state: A–LIG most frequent scenarios; B–Early Holocene most frequent scenarios.

To evaluate the role, visibility and impact of hunter-gatherers’ fires on vegetation, we quantified the number of grid cells affected by each agent across the most frequent scenarios. The parameter values, selected based on the mode of the generated parameter distributions for each time window (Fig 8), are detailed in S6 Table in S1 File.

The mean number of modifications by climate, megafauna, natural and human-induced fires is shown in Fig 8. Climate had a greater influence on PFT distribution (on average 62% of grid cells during the LIG and 72% of grid cells during the Early Holocene) compared to its impact on vegetation openness (9% during the LIG, 35% during the Early Holocene). A consistent trend from the LIG to the Early Holocene is the declining role of megafauna plant consumption, although it remained a significant factor for vegetation openness (77% during the LIG, and 57% during the Early Holocene), but less so for PFT distribution (31% during the LIG and 1% during the Early Holocene). Meanwhile, the visibility of human impact increased. Neanderthals initiated visible changes on a continental scale, though these modifications were minimal during the LIG: Neanderthals impacted PFTs in 6% of grid cells and vegetation openness in 14% grid cells. The Neanderthal impact may have been overwritten by climatic fluctuations and megafauna effects, particularly during the LIG simulation runs. During the Early Holocene, vegetation burning by hunter-gatherers became the second most influential agent for PFT distribution after climate, affecting an average of 26% of European landscapes, with a maximum of 47% of grid cells.

## Discussion

### Temporal vegetation dynamics: CARAIB vs REVEALS

It is important to emphasize that CARAIB and REVEALS reconstruct regional vegetation in different ways, which naturally leads to some divergence between their outputs [6]. CARAIB is driven by climate forcing and modelled vegetation dynamics. REVEALS is based on transformation of pollen count data into quantitative estimates of regional vegetation cover. Moreover, differences in pollen data availability across grid cells between time periods make direct comparisons challenging. REVEALS reconstructions for the Holocene benefit from broader spatial coverage, whereas estimates for the LIG are largely restricted to regions that were glaciated during the late Saalian (MIS 6) (S2 and S4 Figs in S1 File.) [138]. Aligning REVEALS LIG time windows with specific CARAIB outputs is challenging [23,115]. Additionally, the parameter values for foragers’ impact area and preferences for vegetation openness around campsites during the LIG (Fig 7A, E), obtained via the genetic algorithm, are largely applicable to Central Europe, where most REVEALS estimates are concentrated. As a result, continental-scale CARAIB–REVEALS comparisons for the LIG, as well as extrapolation of LIG HUMLAND results to the entire continent, should be done with caution.

It is important to highlight that different areas across Europe have varying post-depositional processes, preservation conditions, and research histories which introduce additional uncertainty when attempting to generalize conclusions at continental scale [138]. Despite these challenges, our study advances our understanding of the potential dynamics of interglacial landscapes and the role of *Homo* within them, particularly during the Early Holocene, where we obtained more robust results due to the relatively extensive REVEALS coverage (Figs 2, 3, S2 and S4 in S1 File). Additionally, this study represents the first attempt to integrate these and other datasets into a single ABM spanning such an extensive period.

A comprehensive comparison between CARAIB and other climate-based vegetation models lies beyond the scope of this study. A recent comparison of CARAIB, Spatially Explicit Individual Based DGVM (SEIB-DGVM), and ORCHIDEE-DGVM against REVEALS data showed statistically similar results compared to REVEALS on the continental scale [139]. Thus, using only CARAIB in our continental-scale study should not be viewed as a limitation. We emphasize that CARAIB is an established and widely used model in paleoclimatic research [53–55].

While testing the impact of different input parameters on the REVEALS output is beyond the scope of our research, it is important to note that the assumptions of the REVEALS model are explicitly defined, ensuring transparency in the interpretation and evaluation of our results. Several of these assumptions have been tested and validated, and the REVEALS model itself has undergone extensive evaluation across multiple areas across Europe [118,140,141], North America [142], and on a continental scale [58], defining a European scale protocol [58,118]. Thus, we believe our findings provide a reliable basis for addressing the research questions of this study.

The differences between the CARAIB and REVEALS datasets remain consistent between the LIG and Early Holocene, except for 11,700–10,200 BP (Fig 5). This exception may be partly attributed to the glacial/interglacial cycle affecting the late arrival of some trees [143,144]. Because of this, distinguishing climate influences on vegetation from other processes is particularly challenging for 11,700–10,200 BP. Therefore, we did not conduct HUMLAND runs for this period (refer to Supporting Information for further clarifications).

The overall similarity in the degree of difference between CARAIB and REVEALS for the Early Holocene and the LIG likely reflects their comparable vegetation development and similar or slightly higher annual LIG temperatures relative to the present interglacial [23]. However, ecosystem dynamics and role of different factors in it varied between these periods, as shown by HUMLAND’s impact quantifications (Fig 8). These differences may be due to discrepancies between the LIG and the Holocene: LIG higher eustatic sea level, variations in insolation [23], shifts in megafauna composition [19], and differences in *Homo* populations.

### HUMLAND scenarios with and without human-induced vegetation burning

Without fires, including natural ones, it is nearly impossible to produce HUMLAND scenarios with vegetation outcomes similar to REVEALS (Table 3). While HUMLAND outputs similar to pollen-based estimates can be generated using natural fires alone, without anthropogenic burning, the likelihood of such scenarios is low (Table 3).

These results indicate that the inclusion of fires set by hunter-gatherers is necessary to consistently generate outputs comparable to REVEALS. Thus, megafauna and climate alone were likely not the only factors shaping vegetation dynamics in Europe, not just during the Early Holocene–as indicated by the first HUMLAND results [6]–but also during the LIG. When fires, particularly human-induced burning, are included in our genetic algorithm experiments, most of the generated outputs align with REVEALS (Table 4), suggesting that fires and particularly anthropogenic fires could have played an important role in European interglacial ecosystems.

The identified importance of fires during the Holocene aligns with findings from other studies, which show an increase in biomass burning in the Early Holocene [20,145]. However, reconstructing the dynamics of fire on a continental scale for the LIG and comparing it to the Early Holocene is challenging due to the limited availability of LIG proxy data [146]. Current estimates indicate that biomass burning was generally more widespread during interglacial phases compared to glacial periods, highlighting the importance of fires in shaping interglacial landscapes–a finding consistent with our results [146,147]. Fire-related patterns during both periods can exhibit similarities due to overall similar vegetation dynamics between the LIG and the Holocene [19,23]. On the other hand, some studies suggest that fire activity may have been more widespread during the Early Holocene than in the LIG [20,147], whereas other regions experienced higher fire frequencies during the LIG [148]. In addition, archaeological evidence points to the importance of fire in locations occupied by LIG Neanderthals [27,149].

The PCA and PCC results indicate that each HUMLAND parameter uniquely contributes to scenarios involving anthropogenic fires (S5 Fig; S3 and S4 Tables in S1 File), making it difficult to identify the most influential parameters or their combinations for overall ecosystem functioning. At the same time, these results showed that the “Hunting_pressure” parameter had a smaller impact during the LIG compared to the Early Holocene (S3 and S4 Tables in S1 File). The following section examines how Neanderthals and Mesolithic humans impacted herbivore plant consumption via assessment of the generated values for this parameter.

### Human–megafauna interaction

To reach REVEALS estimates without anthropogenic burning, HUMLAND hunter-gatherers had to decrease megafauna plant consumption by 20–25% during the LIG and by 80–90% during the Early Holocene (Fig 6). Experiments with anthropogenic fires showed that humans could reduce megafauna plant consumption by 0–39% during the LIG, and by 0–82% during the Early Holocene (Fig 7G, H). Without reducing animal impact through hunting, the simulated vegetation openness would be different than what is shown in the REVEALS data.

Despite lower hunting pressure values in the LIG compared to the Early Holocene, hunting during the LIG was likely important, given the larger megafauna population size before 100,000 BP [89] and emerging evidence for early pre-sapiens megafauna extinctions [90]. In addition, solid evidence suggests that Neanderthals were top carnivores, obtaining protein and fat from terrestrial animals, though not exclusively [150,151]. Neanderthals hunted various animals, including reindeer (*Rangifer tarandus*), horses (*Equus*), larger species such as bovids (Bovidae) and rhinoceros (*Stephanorhinus*) [150,151]. Recent studies have confirmed that Neanderthals also hunted the largest Pleistocene mammals, straight-tusked elephants, and possibly engaged in large-scale collective subsistence activities [152]. This aligns with growing evidence that the largest herbivores were generally preferred [98,153]. Additionally, it is suggested that Neanderthals exhibited animal exploitation practices comparable to those of (sub-)recent foragers [94,150,154,155]. In some cases, local–regional reduction or extinction of animal populations appears to have occurred before the widespread presence of *Homo sapiens* [95,98,156].

HUMLAND scenarios indicate that even in absence of anthropogenic burning, foragers still played a crucial role in vegetation change, albeit indirectly through hunting, which led to a decline in megafauna plant consumption. Thus, interglacial landscapes could have been indirectly affected by *Homo* even without or with reduced anthropogenic burning. However, scenarios without human-induced fires are probably less likely, as suggested by archaeological evidence for fire use from Neanderthal and Mesolithic contexts [4].

### Neanderthal and Mesolithic human impacts on vegetation

By integrating the genetic algorithm in our study, we substantially expanded our ability to generate and explore a diverse range of HUMLAND scenarios. This approach allowed us to efficiently navigate through potential outcomes, providing insights into the complex interactions between humans and the environment. As shown in Table 3, even with relatively good Holocene REVEALS coverage (Figs 2D, 3D), most of the HUMLAND scenarios without human-induced fires fail to produce outputs comparable to REVEALS estimates, particularly for the distribution of dominant PFTs. This result underscores the importance of anthropogenic activities, particularly burning by foragers, for European vegetation dynamics.

#### Preferences for vegetation openness around campsites.

The relevance of human-induced fires for both study periods is further supported by the values derived for the “Openness_criteria_to_burn” parameter which determines the decision-making process of hunter-gatherer groups regarding vegetation burning in a grid cell (Figs 7A, B). These results showed that Neanderthals and Mesolithic humans had similarities in preferences for vegetation openness around their campsites and for starting fires based on surrounding vegetation density. In PFT distribution scenarios both LIG and Early Holocene foragers often burnt areas which were 45–78% open. This suggests that both groups engaged in fire practices across a diverse range of landscapes, including areas that were already relatively open (up to 78%).

On the other hand, scenarios generated for vegetation openness showed clear differences between Mesolithic and Middle Paleolithic strategies. Our results indicate that in most cases Mesolithic humans engaged in burning activities across a broad range of vegetation openness (36–69%). This suggests that these groups may have implemented burning practices across both relatively open and closed areas. Conversely, Neanderthals, in the majority of vegetation openness scenarios, engaged in burning of primarily relatively dense areas (23–48% open).

The observed differences in parameter values for vegetation openness scenarios may be attributed to variations in megafauna influence on vegetation during the study periods. Given the stronger impact of herbivory on vegetation–especially on openness (Fig 8)–during the LIG compared to the Holocene, resulting from larger megafauna populations and differences in community composition, Neanderthals likely needed fewer burning events to achieve vegetation openness around their campsites similar to that preferred by Mesolithic populations. Based on this interpretation of the modelling results, both Mesolithic hunter-gatherers and Neanderthals must have had the ability to alter the vegetation around their campsites, and both groups could burn landscapes relatively often if necessary. The extent of this modification likely depended on their specific subsistence activities, and the initial vegetation openness within the occupied area.

#### Vegetation burning range size around campsites.

Modelling results indicate that the size of the area impacted by foragers remained relatively consistent (~30–40 km around campsites) across both periods for tree dominance scenarios (Fig 7E). For vegetation openness scenarios matching REVEALS data, Neanderthals influenced slightly smaller areas (~20 km), while Mesolithic humans impacted larger areas (~20–30 km) at the beginning of the Holocene, with their influence becoming more localized (~10 km) by the end of the Early Holocene (Fig 7F).

Thus, both Neanderthal and Mesolithic populations showed similarities in their spatial impact patterns in the tree dominance scenarios. Openness scenarios revealed both differences and similarities: Mesolithic humans demonstrated flexible spatial strategies, typically impacting smaller areas (~10 km) but also influencing areas comparable in size to those affected by Neanderthals.

#### Potential minimal population size estimates.

Although estimating *Homo* population sizes is beyond the scope of the current ABM [6], our modelling results may inform on minimal population sizes of European hunter-gatherers. This is because HUMLAND only includes groups that use fire, and not the entire population.

To produce possible scenarios with output similar to the pollen-based vegetation cover, the mean estimated number is 1936–3266 groups for the LIG and 2243–2895 groups for the Early Holocene (Fig 7C, D). Drawing upon the average documented group size of 25 among historical hunter-gatherer societies [157], our modelling suggests that during the Early Holocene, Europe may have had a minimum population ranging from 56,000–72,000 individuals between 10,200 and 8200 BP. These estimates are consistent with the outcomes of the first HUMLAND application [6]. Regarding the LIG minimal population size estimates, HUMLAND indicates that 48,000–82,000 individuals were required to match REVEALS.

It is challenging to compare our minimal population size estimates with other existing data or to directly evaluate the HUMLAND results from both periods. Since HUMLAND can only estimate potential minimal population size, our Early Holocene estimates are generally lower than the currently available continental-scale estimates, which range from approximately 80,000–180,000 [49,50] and 52,000–1,111,000 [51]. Our minimum estimate of 56,000 is consistent with the lower bound of the latter range.

The HUMLAND minimum population size estimates for the LIG are comparable to those for the Early Holocene. Our LIG values generally align with and slightly exceed the only available census estimates for Neanderthals, which suggest a broad range of 5000–70,000 individuals without specifying particular geographic regions or temporal intervals within Neanderthal history [41]. It has been suggested that the Neanderthal population may have increased during some phases [52], such as the LIG, due to higher ungulate populations and an abundance of plant resources under favorable interglacial conditions [41]. Therefore, it is difficult to support the widely-held assumption that the overall hunter-gatherer population size during the Early Holocene exceeded that of the LIG–an assumption often interpreted as implying a greater impact on vegetation by Holocene foragers [28,30]. The available distribution patterns of LIG archaeological sites are likely incomplete, determined by large-scale geomorphological processes and research bias, rendering LIG sediments difficult to access [33,138,158]. Unlike Mesolithic sites, the LIG archaeological evidence has undergone a complete glacial–interglacial cycle, which rendered most surviving sites inaccessible due to the deposition of covering layers [33]. Furthermore, most of the Mesolithic evidence consists of (surface) flint scatters that can be attributed to this phase based on typological characteristics alone [33]. Conversely, there are no distinctive stone tools produced by Neanderthals that can be attributed specifically to the LIG. Instead, site identification relies on a combination of stratigraphic data and multiple paleoenvironmental proxies, hence requiring a taphonomic setting that is only rarely encountered [33].

Thus, our modelling exercise suggests that the number of groups required to align the HUMLAND output with REVEALS is comparable for both the LIG and the Mesolithic. As we can only provide minimum estimates for both populations, this finding does not exclude the possibility that the census size of the two populations did differ, potentially being higher in one of the study periods. However, we currently lack sufficient data to determine this definitively.

An additional complexity in assessing the HUMLAND population size estimates and the vegetation openness preference values is the absence of thunderstorm frequency data for the study periods. Instead, we used modern values [133], which may not accurately reflect past environments. Distinguishing between natural fires and human-induced burning is often challenging in paleoenvironmental proxies [4]. This uncertainty suggests that the obtained minimal population estimates and vegetation openness degree to start fires should, to some extent, be adjusted, if thunderstorm frequency was different during the LIG and the Early Holocene than today. While lightning is the main source of natural fires [159,160], the occurrence and spread of fire also depend on additional factors (e.g., fuel accumulation and moisture, weather and seasonal changes). HUMLAND incorporates these aspects to some extent: different PFTs have varying probabilities of fire ignition, and megafaunal activity and fires reduce available fuel. Some important variables such as wind patterns and seasonal climate variability are outside the temporal and spatial focus of our study. Nevertheless, any increase in the contribution of natural fires to vegetation changes would likely be limited, given the overall comparable climatic conditions between the Holocene and the LIG.

#### Visibility of anthropogenic burning on continental level.

To properly interpret the calculated extent of modifications done by each agent (Fig 8), it is crucial to consider that HUMLAND records only the last agent responsible for the final vegetation change. Within a single simulation step, the model initiates impacts on vegetation in the following order: anthropogenic vegetation burning, natural fires, megafauna plant consumption, and in the subsequent step, vegetation regeneration due to climatic effects for grid cells previously affected by fires or animals (Fig 4). This ordering means that anthropogenic impacts (earlier in the sequence) may be overwritten by subsequent events. While the model effectively captures human-induced fire effects [6], human impacts can be masked by later processes, leading the model to reflect only the minimal detectable human influence, rather than the full extent of anthropogenic impacts on vegetation.

The percentages of grid cell modifications by each agent (Fig 8) demonstrate that megafauna influences vegetation openness across numerous grid cells within HUMLAND. It is important to emphasize that, at each simulation step, herbivores do not reduce vegetation by more than 1% on any given grid cell. This calculation is based on the combination of CARAIB NPP and the potential maximum megafauna plant consumption (for further details see the Materials and methods section). Despite this modest per-step reduction, herbivory affects a substantial number of grid cells at the continental scale, and through its cumulative effect, replaces the first dominant PFT in approximately 30% of grid cells during the LIG and in 1% during the Early Holocene, reflecting differences in megafauna populations between these periods. Overall, the quantitative impact of herbivory remains lower than that of a fire event in a single simulation step, as fire immediately diminishes all vegetation within the affected grid cells in HUMLAND.

The HUMLAND results show the megafauna’s influence on the overall vegetation structure during the LIG combined with climatic effects playing a key role in transforming European vegetation (Fig 8A). However, scenarios without human-induced fires (Table 3) indicated that megafauna and climate alone did not produce results similar to REVEALS especially for the PFT distribution. This underscores the role of both Neanderthals and Mesolithic humans in shaping interglacial vegetation dynamics. The mean percentage of grid cells modified by Neanderthals is relatively low: on average 6% for PFT distribution and 14% for vegetation openness (Fig 8A). Nonetheless, Neanderthal impact remains detectable and represents an important component of overall interglacial ecosystem dynamics. By initiating vegetation changes that made certain areas more appealing to animals, Neanderthals may have enhanced herbivore impacts in recently burnt regions. However, the visibility of Neanderthal impacts may be obscured by climatic fluctuations and subsequent megafauna activity.

During the Early Holocene, megafauna continued to be a key driver of vegetation openness (Fig 8B). Despite this significant influence, herbivores had minimal impact on PFT distribution (only 1% on average, Fig 8B). Mesolithic humans were the second most influential factor after climate in shaping PFT distribution through fire use, consistent with earlier HUMLAND findings [6], even with the improved representation of megafauna plant consumption in HUMLAND 2.0. HUMLAND results showed that, unlike megafauna, Mesolithic humans could open up vegetation and even completely replace shrubs and trees with bare ground, where herbs regrew. This ability allowed Mesolithic humans to transform approximately 26% of grid cells on average, reaching a maximum of 47% in PFT distribution, and to alter vegetation openness in 8% of grid cells on average, with a maximum of 14%. These findings indicate that human agency played a substantial role in shaping European landscapes, already before the emergence of agriculture (Fig 8B; Tables 3 and 4).

## Conclusion

By combining the spatially explicit HUMLAND ABM with a genetic algorithm to manipulate parameter values we were able to generate scenarios of early human-induced vegetation changes that match pollen reconstructions during the LIG and the Early Holocene in Europe. Our findings suggest that hunter-gatherers had a substantial impact on interglacial vegetation through the use of fire. The simulation outcomes suggest that human activities may have affected approximately 26% of PFT distributions, with a potential maximum of 47%, and on average, 8% of the vegetation openness, with a maximum of 14%, across the European landscape before the emergence of agriculture. HUMLAND outputs showed that megafauna, natural fires, and climatic fluctuations alone were insufficient to produce the pollen-based vegetation reconstructions, highlighting the importance of human agency in altering vegetation cover. These findings align with existing ethnographic studies on hunter-gatherer impact on landscapes, as well as archaeological evidence from Neanderthal and Mesolithic case studies.

Our results demonstrate that climate and especially megafauna played an impotant role in vegetation transformation during both the LIG and the Mesolithic, with a stronger effect of megafauna in the LIG. At the same time, foragers in both periods contributed to vegetation changes through fire use. In scenarios where human-induced burning was minimal or absent, both Neanderthals and Mesolithic humans still shaped landscapes indirectly by hunting large herbivores, thereby reducing their browsing and grazing pressure on vegetation. Without hunting pressure, vegetation in HUMLAND would have been different (likely more open during the LIG) than pollen-based estimates suggest.

Our modelling exercise suggested that Neanderthals and Mesolithic humans shared similarities in their impact. Scenarios generated using the genetic algorithm showed that both groups influenced similarly sized areas around their campsites, had similar preferences for vegetation openness, and a comparable number of groups was requred to align HUMLAND model outputs with REVEALS data.

Future research should address gaps in the archaeological and paleoecological record identified by our study and expand our approach to other time periods and continents by incorporating more CARAIB–REVEALS comparisons in the HUMLAND ABM. The American continent is of particular interest, as the late arrival of *Homo sapiens* there allows for comparisons between landscapes with and without human impact. To enhance the precision and reliability of future modelling exercises on early human impact on landscapes via improving the quantity of proxy-based reconstructions, such as REVEALS, necessitates an expansion in the geographic coverage and density of sites from which proxies are obtained. Furthermore, modelling approaches and setups used in generating datasets that could be included in models like HUMLAND require refinements to minimize inherent biases and limitations (e.g., vegetation response to deglaciation within dynamic vegetation models). Local-scale research holds high relevance for studying past human-environment interactions to test whether patterns observed at the continental level are also visible at finer scales.

## Supporting information

S1 File Supporting information–Includes supplementary figures, tables and additional details on the modelling setup, Pearson correlation coefficients, principal component analysis, and the CARAIB–REVEALS comparison. (PDF)

S2 File Generated scenarios–Data file. (ZIP)
