# Supplementary material for: On the ecological impact of prehistoric hunter-gatherers in Europe: Early Holocene (Mesolithic) and Last Interglacial (Neanderthal) foragers compared
Source: PLoS One. 2025 Oct 22;20(10):e0328218. doi: 10.1371/journal.pone.0328218 (PMC12543167; doi:10.1371/journal.pone.0328218)
Supplement: S1 File — (PDF) [file pone.0328218.s001.pdf]

Supporting Information

On the ecological impact of prehistoric hunter-gatherers in Europe: Early Holocene (Mesolithic) and Last Interglacial (Neanderthal) foragers compared

Anastasia Nikulina, Anhelina Zapolska, Maria Antonia Serge, Didier M. Roche, Florence Mazier, Marco Davoli, Elena A. Pearce, Jens-Christian Svenning, Dave van Wees, Ralph Fyfe, Katharine MacDonald, Wil Roebroeks, Fulco Scherjon

*Corresponding author. Email: a.nikulina@arch.leidenuniv.nl; anastasia.nikulina@durham.ac.uk; nikulina1302@gmail.com

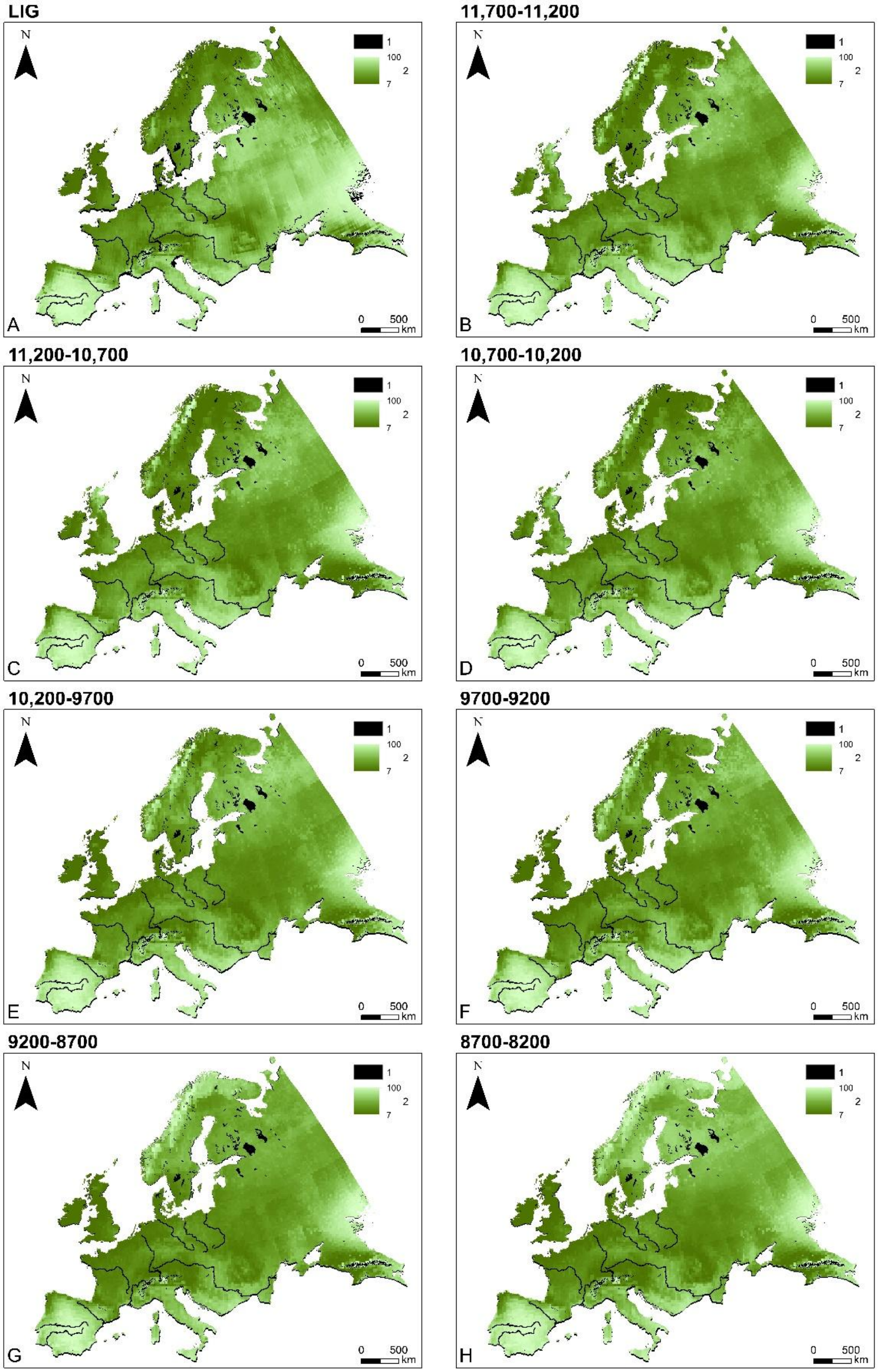

S1 Fig. CARAIB vegetation openness for the Last Interglacial (A), 11,700–11,200 BP (B), 11,200–10,700 (C), 10,700–10,200 (D), 10,200–9700 (E), 9700–9200 (F), 9200–8700 (G), 8700–8200 BP (H). Legend: 1–No data, 2–Vegetation openness (in %).

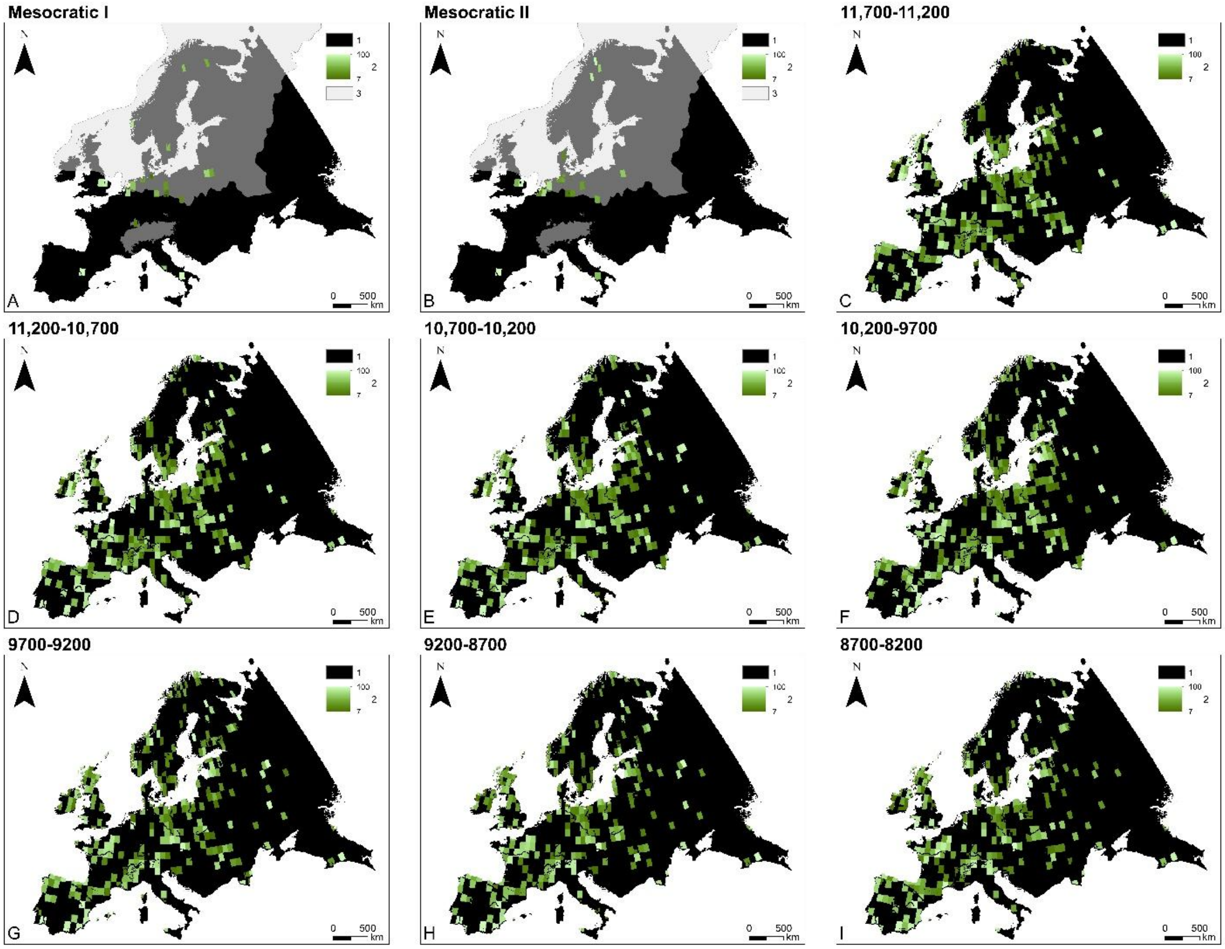

S2 Fig. REVEALS vegetation openness for Mesocratic I (A), Mesocratic II (B), 11,700–11,200 BP (C), 11,200–10,700 (D), 10,700–10,200 (E), 10,200–9700 (F), 9700–9200 (G), 9200–8700 (H), 8700–8200 BP (I). Legend: 1–No data, 2–Vegetation openness (in %); 3–The northern European and Alpine Saalian glaciation (after 1,2).

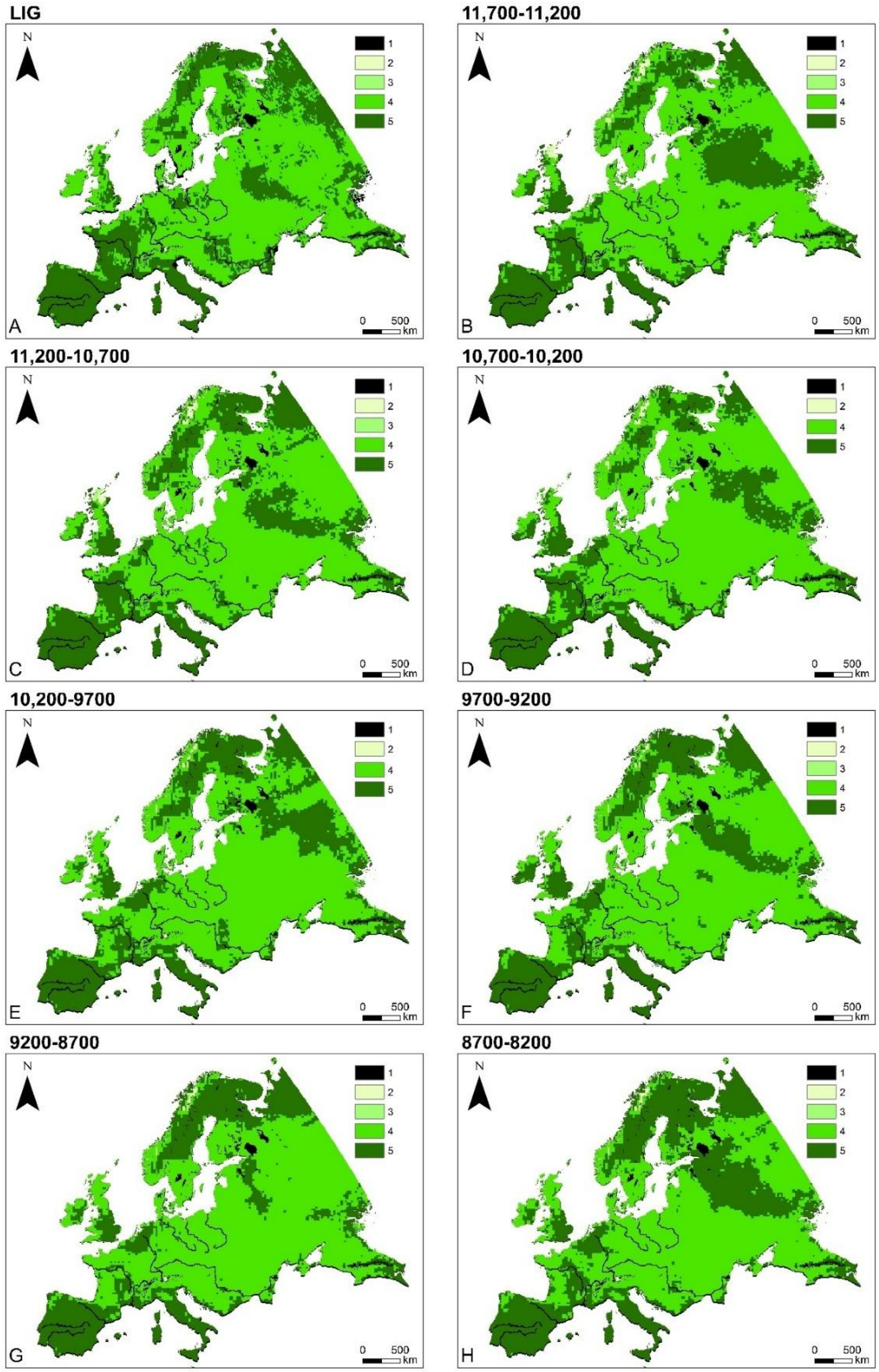

S3 Fig. CARAIB distribution of the first dominant PFTs for the Last Interglacial (A), 11,700–11,200 BP (B), 11,200–10,700 (C), 10,700–10,200 (D), 10,200–9700 (E), 9700–9200 (F), 9200–8700 (G), 8700–8200 BP (H). Legend: 1–No data, 2–Herbs, 3–Shrubs; 4–Broadleaf trees; 5–Needleleaf trees.

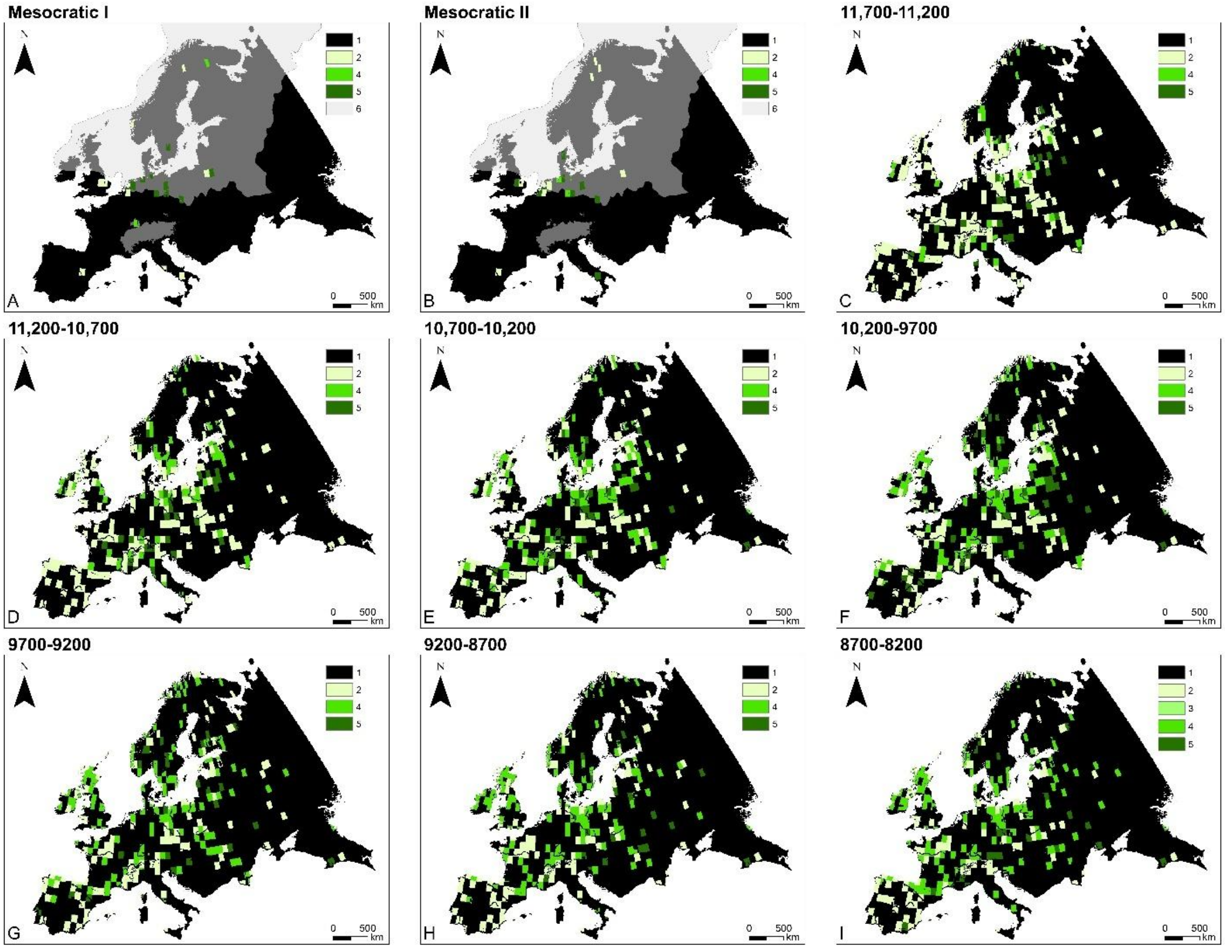

S4 Fig. REVEALS distribution of the first dominant PFTs for Mesocratic I (A), Mesocratic II (B), 11,700–11,200 BP (C), 11,200–10,700 (D), 10,700–10,200 (E), 10,200–9700 (F), 9700–9200 (G), 9200–8700 (H), 8700–8200 BP (I). Legend: 1–No data, 2–Herbs, 3–Broadleaf trees; 4–Needleleaf trees, 5–The northern European and Alpine Saalian glaciation (after 1,2).

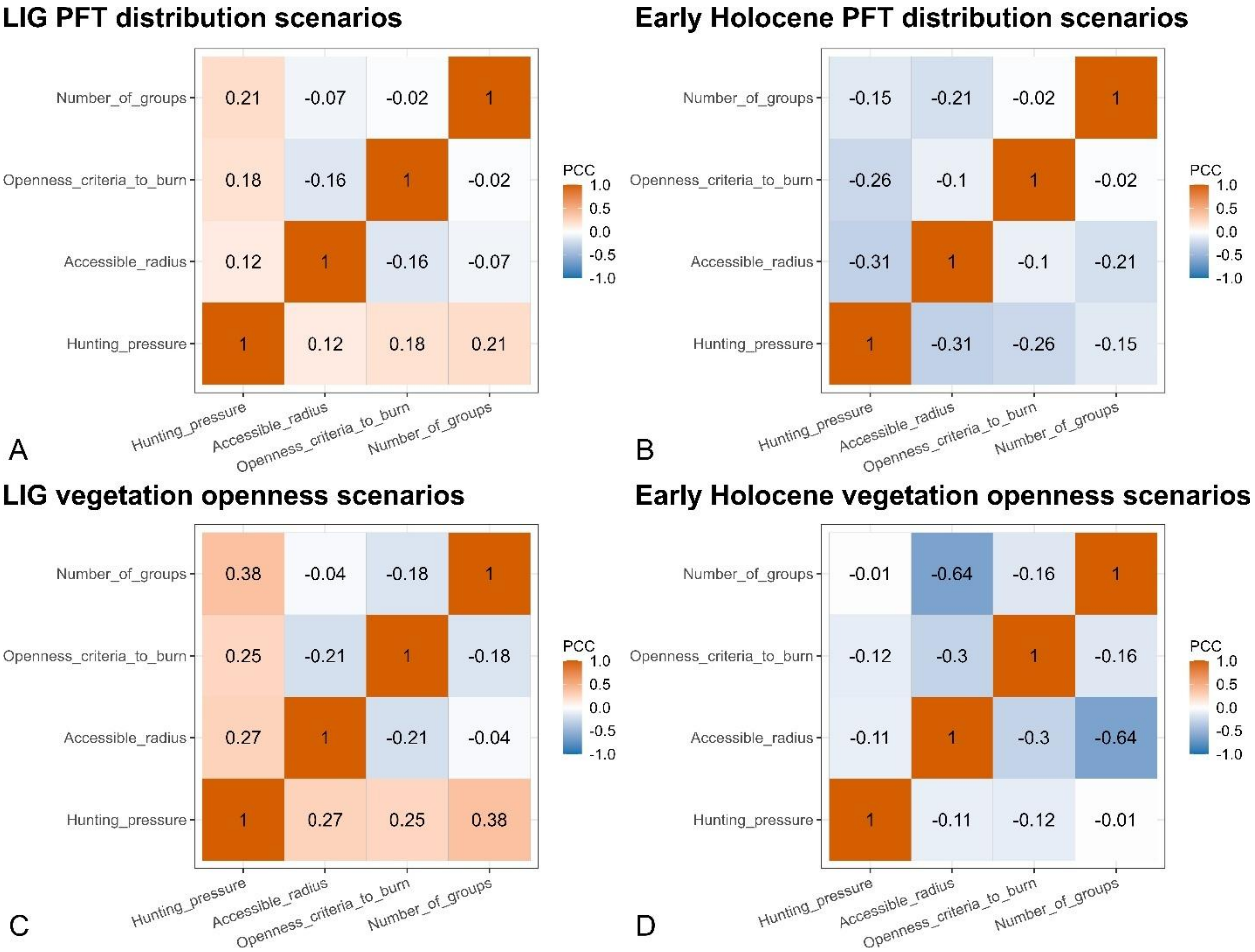

S5 Fig. Correlation matrices and Pearson correlation coefficients (PCC) between variables of the possible scenarios for LIG (A) and Early Holocene (B) tree distribution scenarios; LIG (C) and Early Holocene (D) vegetation openness scenarios. The experiments include the combined direct impact of all agents on vegetation: anthropogenic and natural fires, climatic impact and megafauna plant consumption. The darkest blue indicates the strongest negative correlation between the "Number_of_groups" and "Accessible_radius" parameters within the Early Holocene vegetation openness scenarios. Lighter colours represent either absent/low or modest correlations for the other parameters.

S1 Table. PFTs used in ABM (HUMLAND PFTs) and correspondence between CARAIB PFTs and REVEALS plant taxa (after 3).

CARAIB PFTs	Plant taxon / pollen morphological types	HUMLAND PFTs
Needle-leaved evergreen boreal/temp cold trees<br>Needle-leaved evergreen meso mediterranean trees<br>Needle-leaved evergreen subtropical trees<br>Needle-leaved evergreen supra mediterranean trees<br>Needle-leaved evergreen temperate cool trees<br>Needle-leaved summergreen boreal/temp cold trees<br>Needle-leaved summergreen subtropical swamp trees	*Abies*<br>*Picea*<br>*Pinus*<br>*Juniperus*	Needleleaf trees
Broadleaved evergreen meso mediterranean trees<br>Broadleaved evergreen subtropical trees<br>Broadleaved evergreen thermo mediterranean trees<br>Broadleaved evergreen tropical trees<br>Broadleaved raingreen tropical trees<br>Broadleaved summergreen boreal/temp cold trees<br>Broadleaved summergreen temperate cool trees<br>Broadleaved summergreen temperate warm trees	*Alnus*<br>*Betula*<br>*Carpinus betulus*<br>*Carpinus orientalis*<br>*Castanea sativa*<br>*Corylus avellana*<br>*Fagus*<br>*Fraxinus*<br>*Phillyrea*<br>*Pistacia*<br>deciduous *Quercus* t.<br>evergreen *Quercus* t.<br>*Salix*<br>*Tilia*<br>*Ulmus*	Broadleaf trees

Broadleaved evergreen boreal/temp cold shrubs<br>Broadleaved evergreen temperate warm shrubs<br>Broadleaved evergreen xeric shrubs<br>Broadleaved summergreen arctic shrubs<br>Broadleaved summergreen boreal/temp cold shrubs<br>Broadleaved summergreen temperate warm shrubs<br>Subdesertic shrubs<br>Tropical shrubs	*Buxus sempervirens*<br>*Calluna vulgaris*<br>Ericaceae	Shrubs
C3 herbs ("dry")<br>C3 herbs ("humid")<br>C4 herbs	Amaranthaceae/Chenopodiaceae<br>*Artemisia*<br>Cerealia t.<br>Cyperaceae<br>*Filipendula*<br>*Plantago lanceolata*<br>Poaceae<br>*Rumex acetosa* t.<br>*Secale cereale*	Herbs

S2 Table. Datasets used in HUMLAND (after 3)

Dataset	Initial data type	Initial spatial resolution/scale	Meaning, units	Source
GTOPO30	Raster	1 km	Digital elevation model, m	https://www.usgs.gov/
WISE	Vector	1:10,000,000	Distribution of large rivers and lakes	https://water.europa.eu/
CARAIB first dominant PFT	Raster	~26 km (0.25°)	PNV: first dominant PFT	http://www.umccb.ulg.ac.be/Sci/m_car_e.html
CARAIB vegetation openness			PNV: vegetation openness (%)	
NPP			PNV NPP (excluding carbon used for respiration), g/m^2	
Megafauna vegetation consumption	Raster	30 km	Potential maximal megafauna vegetation consumption (i.e., metabolization of NPP), kg/km^2 (converted to g/m^2)	4,5
REVEALS first dominant PFT	Vector	~100 km (1°)	Pollen-based first dominant PFT	6
REVEALS vegetation openness			Pollen-based past vegetation openness (relative %)	
REVEALS vegetation			Standard errors for estimates of pollen-	

openness standard errors			based past vegetation openness	

S3 Table. PCA results for successful genetic algorithm outputs aiming to minimize the HUMLAND–REVEALS difference in mean percentage of grid cells dominated by trees. The experiments include the combined impact of all agents on vegetation: anthropogenic and natural fires, hunting, climatic impact and megafauna plant consumption.

Time windows	Variables	Openness_criteria_to_burn	Hunting_pressure	Number_of_groups	Accessible_radius
Mesocratic I	Comp. 1 (54.2%)	0.54	0.07	0.36	-0.74
	Comp. 2 (26.1%)	-0.63	0.24	0.72	-0.08
Mesocratic II	Comp. 1 (46.7%)	0.78	0.04	-0.44	-0.42
	Comp. 2 (36.5%)	0	0.06	0.69	-0.71
10,200–9700 BP	Comp. 1 (44.7%)	-0.31	0.17	-0.67	-0.63
	Comp. 2 (36%)	-0.18	0.82	-0.17	-0.5
9700–9200 BP	Comp. 1 (48.3%)	-0.31	0.48	-0.68	0.44
	Comp. 2 (30.4%)	-0.01	0.69	0.02	-0.72
9200–8700 BP	Comp. 1 (51.2%)	-0.51	0.65	0.33	-0.43
	Comp. 2 (28%)	-0.66	0	-0.05	0.74
8700–8200 BP	Comp. 1 (47.8%)	-0.27	0.58	0.31	0.69
	Comp. 2 (33.9%)	-0.38	0.53	-0.69	0.29

S4 Table. PCA results for the successful genetic algorithm outputs aiming to minimize the HUMLAND–REVEALS difference in mean vegetation openness. The experiments include the combined impact of all agents on vegetation: anthropogenic and natural fires, hunting, climatic impact and megafauna plant consumption.

Time windows	Variables	Openness_criteria_to_burn	Hunting_pressure	Number_of_groups	Accessible_radius
Mesocratic I	Comp. 1 (58.8%)	-0.74	0.09	0.40	0.51
	Comp. 2 (29.4%)	-0.02	0.11	-0.75	-0.65
Mesocratic II	Comp. 1 (45.1%)	0.77	0.01	-0.17	-0.61
	Comp. 2 (43.7%)	-0.22	0.24	-0.79	-0.51
10,200–9700 BP	Comp. 1 (59.9%)	0.16	0.25	0.63	-0.71
	Comp. 2 (27%)	-0.84	0.44	0.24	0.17
9700–9200 BP	Comp. 1 (50.3%)	-0.16	0.39	0.64	-0.62
	Comp. 2 (33.8%)	-0.83	0.1	0.2	0.49
9200–8700 BP	Comp. 1 (61.7%)	-0.14	0.09	0.67	-0.72
	Comp. 2 (21.4%)	-0.85	0.47	0.19	0.06
8700–8200 BP	Comp. 1 (56%)	0.12	0.05	0.67	-0.72
	Comp. 2 (27.8%)	-0.81	0.52	0.22	0.1

S5 Table. Mean, mode and standard deviation (SD) for parameter values obtained via genetic algorithm with outputs similar to REVEALS. The distribution of parameter values is in Fig. 7 of the main body of the article.

Time windows	Parameters and experiment types																							
	Openness_to_burn						Hunting_pressure						Number_of_groups						Accessible_radius					
	Trees			Openness			Trees			Openness			Trees			Openness			Trees			Openness		
	Mean	SD	Mode	Mean	SD	Mode	Mean	SD	Mode	Mean	SD	Mode	Mean	SD	Mode	Mean	SD	Mode	Mean	SD	Mode	Mean	SD	Mode
LIG	77	14	55<br>77<br>78	49	23	48	24	29	4	34	13	21<br>22<br>23<br>24<br>25<br>27<br>31<br>39	3266	591	3323	1936	1136	563	3	1	3	2	1	1
Mesocratic I	79	13	78	48	22	23	27	33	4	33	15	21<br>22<br>23	3381	488	3323	2044	1122	139<br>1087<br>2017<br>2515	3	1	3	2	1	2
Mesocratic II	75	15	55<br>77	50	24	48	22	25	0<br>1	35	11	24<br>27<br>31<br>39	3156	657	2943	1830	1140	563	3	1	3	2	1	1
Ea	71	17	45	60	22	36	48	27	31	34	26	4		691	3575		957		3	1	4	3	1	1

			47<br>57<br>59<br>69			38<br>42<br>62<br>69							2895			2243		1089						
10,200–9700 BP	76	15	57	61	23	36<br>62	45	25	31	40	29	1<br>6<br>10<br>44<br>74<br>82	3071	462	3161	2090	759	1329	3	1	4	3	1	2
9700–9200 BP	71	15	65	58	22	38	51	25	24<br>30<br>45<br>60<br>67	43	26	35<br>43<br>57	3117	543	3115	2074	897	1188<br>3453	3	1	4	2	1	3
9200–8700 BP	68	20	45<br>59	60	21	38	51	29	43	28	23	4	2550	911	3453	2342	1085	1627	3	1	4	2	1	1
8700–8200 BP	68	17	47	62	23	42	45	29	0<br>3	25	21	21	2843	608	2490	2466	998	1079<br>1460<br>2901<br>3315	3	1	4	2	1	1

						69			38									3336						

S6 Table. Details of HUMLAND runs conducted to track the extent and visibility of modifications done by each agent.

Time windows	Parameter values							
	Openness_to_burn		Hunting_pressure		Number_of_groups		Accessible_radius	
	Trees	Openness	Trees	Openness	Trees	Openness	Trees	Openness
Mesocratic I	81	29	0	21	3323	139	3	4
		29		23		1091		2
		37		26		2017		1
		28		21		2497		3
Mesocratic II	92	33	10	30	2943	563	4	4
10,200–9700 BP	87	47	42	11	3161	1329	4	5
9700–9200 BP	74	72	52	42	3123	1191	4	4
		80		74		3375		1
9200–8700 BP	81	41	75	6	3450	1627	2	1
8700–8200 BP	71	77	14	16	2488	1079	5	3
		72		9		1460		2
		92		10		2901		1
		62		7		3315		1

Paleoenvironmental modelling setup

The potential natural vegetation (PNV) simulations in this study were conducted using a modelling framework that combines ILOVECLIM climate model, and VECODE and CARAIB vegetation models. Below, we detail the configurations and roles of each model.

ILOVECLIM: Paleoclimate Simulation

Climate simulations were performed with the ILOVECLIM Earth System model of intermediate complexity (7), revised by Roche (8) and further expanded by Quiquet et al. (9). The applied version of iLOVECLIM includes the following: the atmospheric model, ECBilt (10), the sea-ice ocean component, CLIO (11), and the reduced-form dynamic global vegetation model (DGVM), VECODE (12). These components are used to simulate climate.

iLOVECLIM operates on a relatively low spatial resolution T21 grid (5.625 ˚ latitude/longitude), which in the current study is increased to 0.25 ˚ latitude/longitude through the use of the online interactive downscaling method embedded in iLOVECLIM, first described by Quiquet et al. (9) and tested within the current modelling setup by Zapolska et al. (13).

We applied iLOVECLIM to simulate evolution of the climate during the Holocene and LIG through a set of transient runs. Holocene transient run was resampled to a time step that correspond to REVEALS time windows (TWs): time windows between the year 6200 BP and the year 700 BP were assigned at 500 years temporal resolution, following by fixed time windows at 350 (700–350 BP), 250 (350–100 BP), and 165 (2015 CE–1850). To simulate climate during the Last Interglacial (LIG) we first performed a transient iLOVECLIM run over the whole LIG and identified periods with high forest fraction in VECODE outputs: 120,000 BP, 124,000 BP, and 128,000 BP. For these three periods we performed equilibrium climate simulations, which were used to drive the CARAIB model. The transient experiments were initialised with states derived from 3000-year long equilibrium simulations at 11,700 BP (early Holocene) and 129,000 BP (early LIG).

For all simulations, we used the following boundary conditions: standardised boundary conditions for palaeoclimate simulations, provided by the Palaeoclimate Modelling Intercomparison Project Phase 4 (PMIP-4) (14), astronomical parameters from Berger (15), greenhouse gas levels (16,17), ice sheets from the GLAC-1D reconstruction (18,19) as well as evolving bathymetry and land-ocean mask coherent with those ice-sheet geometries (with the same methodology as Bouttes et al., 2023; (20).

To further improve reliability of the modelled results in context of intercomparison with pollen data, we applied the CDF-t bias correction technique (21) to correct biases of iLOVECLIM modelled results (13).

VECODE: Dynamic Vegetation Modelling

To provide a necessary climate-biomass feedback loop for the climate simulations we used a reduced-form DGVM VECODE (12). VECODE simulates eco-physiological characteristics of vegetation and soil dynamics in a manner necessary for climate models of intermediate complexity. Vegetation in VECODE DGVM is described using two plant functional types (PFTs): trees and grass (with bare ground as a dummy type).

VECODE dynamics is coupled with atmospheric and oceanic modules of iLOVECLIM at an annual timestep, which simulates plant and soil behaviors necessary for accurately simulating the first-order vegetation-climate feedback in iLOVECLIM. However, its level of complexity is not enough to reflect fine-scale changes that are typically attributed to human impact on vegetation. Thus, iLOVECLIM-simulated bias corrected climate was used as an input for CARAIB, a more complex vegetation model.

CARAIB: High-Resolution Vegetation Modelling

CARAIB (CARbon Assimilation In the Biosphere) is a grid-point process-based dynamic vegetation model that operates at a grid size of the provided input data (here 0.25° latitude/longitude). CARAIB is a comprehensive and mechanistic vegetation model that simulates the vegetation dynamics based on its relationship with climatic and soil conditions.

It combines several modules: hydrological budget (22), canopy photosynthesis and stomatal regulation, carbon allocation and plant growth (23), heterotrophic respiration and litter/soil carbon dynamics, plant competition and biogeography. CARAIB outputs used in this ABM include distribution of fractions of 26 PFTs (PNV distribution), PNV vegetation openness, and potential natural NPP per 26 km × 26 km grid cell.

To simulate the potential natural vegetation during the Holocene we conducted a series of equilibrium runs with the same boundary conditions and spatio-temporal resolution as iLOVECLIM, using its simulated climate as input and obtaining CARAIB-simulated PNV.

REVEALS estimates for LIG provide data for the highest forested period during the LIG without specifying time bounds of such period. Hence, to represent the peak of forest fraction in LIG we performed three equilibrium CARAIB simulations at 120,000 BP, 124,000 BP, and 128,000 BP. These three periods were selected due to their high forested fraction in VECODE outputs (integrated vegetation module within iLOVECLIM climate model). These simulations (not shown) determined that 128,000 BP had the highest forest fraction during the LIG within our setup. The corresponding CARAIB output for this period was thus used in the HUMLAND 2.0 LIG simulations.

Pearson correlation coefficients and Principal component analysis

In Figure S5, the variables within the LIG dataset have both positive and negative correlations, while in the Early Holocene results, correlations are exclusively negative (blue). The magnitudes of the correlation coefficients between parameters are generally modest or low/absent for both LIG (-0.21–0.38) and the Early Holocene (-0.3–0) experiments. Relatively strong correlation (-0.64) is identified between the “Number_of_groups” and “Accessible_area” parameters within the vegetation openness experiments (S5D Fig.).

PCA results show that contribution of some variables to principal components (i.e., new variables that are derived from an original set of variables to reduce the dimensionality of data) vary through time and genetic algorithm experiment group (i.e., minimization of the difference in mean vegetation openness or in percentage of grid cells dominated by trees). The distinct result is that the absolute loadings (i.e., how much a variable contributes to the component) of the “Hunting_pressure” parameter are overall lower for LIG results than for the Holocene runs (S3 and S4 Tables). The absolute loadings of the “Openness_criteria_to_burn” parameter are relatively high for the LIG results regarding PFT distribution (S4 Table). The absolute loadings of this parameter slightly decrease for the dominance of trees experiments in the earlier part of the Early Holocene, and increase again during 9200–8700 BP (S4 Table). The absolute loadings for the “Number_of_groups” and the “Accessible_radius” parameter are relatively high for all time periods (S3 and S4 Tables).

CARAIB–REVEALS comparison for 11,700–10,200 BP

REVEALS showed higher percentages of herbs in comparison with the percentage of trees during 11,700–10,200 BP and the inversion of these values between 10,200–9200 BP (Fig. 3, bottom figure). These observations might be partially explained by the position of these periods within the glacial/interglacial cycle which could entail a late arrival of some tree types (24,25). The duration of postglacial migration lags is unclear. There are suggestions for both relatively short lags of maximally 1500 years, and substantially longer ones including estimates that many plant species have not reached equilibrium with climate even nowadays (26–29). It is also unclear whether the observed species-level lags impact continental-scale distribution of forests (27). Due to that, distinguishing between the potential influences of human activities and climate could be challenging in this context for the 11,700–10,200 BP. In addition, the CARAIB vegetation model used in this study is driven by outputs from an equilibrium iLOVECLIM climate model. In the present setup, both the vegetation and climate models are in equilibrium, and hence do not capture transient changes. ILOVECLIM uses ice sheet data, which then remain static throughout the equilibrium-based simulation. This setup inherently limits representation of several aspects of the Early Holocene, including the transition to warmer conditions in the beginning of the Holocene and the associated soil changes due to deglaciation (transient change in soil composition, texture, and nutrient availability). Thus, we made a deliberate decision not to conduct HUMLAND simulations for 11,700–10,200 BP. We have directed our focus on 10,200–8200 BP and two LIG time windows.

Bibliography

1. Svendsen JI, Alexanderson H, Astakhov VI, Demidov I, Dowdeswell JA, Funder S, et al. Late Quaternary ice sheet history of northern Eurasia. Quat Sci Rev. 2004;23:1229–71.

2. Lehmkuhl F, Nett JJ, Pötter S, Schulte P, Sprafke T, Jary Z, et al. Loess landscapes of Europe – Mapping, geomorphology, and zonal differentiation. Earth Sci Rev. 2021;215.

3. Nikulina A, MacDonald K, Zapolska A, Serge MA, Roche DM, Mazier F, et al. Hunter-gatherer impact on European interglacial vegetation: A modelling approach. Quat Sci Rev. 2024;324.

4. Davoli M., Kuemmerle T, Monsarrat SM, Pacifici M, Crees J, Cristiano A, et al. Recent Sociocultural Changes Reverse the Long-Term Trend of Declining Habitat Availability for Large Wild Mammals in Europe. Diversity and Distributions. 2024;30(12).

5. Davoli M, Monsarrat S, Pedersen R, Scussolini P, Karger DN, Normand S, et al. Megafauna diversity and functional declines in Europe from the Last Interglacial to the present. Global Ecology and Biogeography. 2023;33:34–47.

6. Serge MA, Mazier F, Fyfe R, Gaillard MJ, Klein T, Lagnoux A, et al. Testing the Effect of Relative Pollen Productivity on the REVEALS Model: A Validated Reconstruction of Europe-Wide Holocene Vegetation. Land (Basel). 2023;12(5).

7. Goosse H, Brovkin V, Fichefet T, Haarsma R, Huybrechts P, Jongma J, et al. Description of the Earth system model of intermediate complexity LOVECLIM version 1.2. Geosci Model Dev. 2010;3(2):603–33.

8. Roche DM. δ18 O water isotope in the iLOVECLIM model (version 1.0) - Part 1: Implementation and verification. Geosci Model Dev. 2013;6:1481–91.

9. Quiquet A, Roche DM, Dumas C, Paillard D. Online dynamical downscaling of temperature and precipitation within the iLOVECLIM model (version 1.1). Geosci Model Dev. 2018;11(1):453–66.

10. Opsteegh JD, Haarsma RJ, Selten FM, Kattenberg A. ECBILT: a dynamic alternative to mixed boundary conditions in ocean models. Tellus. 1998;(3):348–67.

11. Goosse H, Fichefet T. Importance of ice-ocean interactions for the global ocean circulation: A model study. J Geophys Res Oceans. 1999;104(C10):23337–55.

12. Brovkin V, Ganopolski A, Svirezhev Y. A continuous climate-vegetation classification for use in climate-biosphere studies. Ecol Modell. 1997;101(2–3):251–61.

13. Zapolska A, Vrac M, Quiquet A, Extier T, Arthur F. Improving biome and climate modelling for a set of past climate conditions : evaluating bias correction using the CDF-t approach. Environmental Research Climate. 2023;2.

14. Kageyama M, Albani S, Braconnot P, Harrison SP, Hopcroft PO, Ivanovic RF, et al. The PMIP4 contribution to CMIP6 - Part 4: Scientific objectives and experimental design of the PMIP4-CMIP6 Last Glacial Maximum experiments and PMIP4 sensitivity experiments. Geosci Model Dev. 2017;10(11):4035–55.

15. Berger A. Long-term variations of daily insolation and Quaternary climatic changes. J Atmos Sci. 1978;35:2362–7.

16. Raynaud D, Barnola JM, Chappellaz J, Blunier T. The ice record of greenhouse gases: a view in the context of future changes. Quat Sci Rev. 2000;19:9–17.

17. Schilt A, Baumgartner M, Blunier T, Schwander J, Spahni R, Fischer H, et al. Glacial-interglacial and millennial-scale variations in the atmospheric nitrous oxide concentration during the last 800,000 years. Quat Sci Rev. 2010;29:182–92.

18. Tarasov L, Peltier WR. Greenland glacial history and local geodynamic consequences. Geophys J Int. 2002;150(1):198–229.

19. Tarasov L, Dyke AS, Neal RM, Peltier WR. A data-calibrated distribution of deglacial chronologies for the North American ice complex from glaciological modeling. Earth Planet Sci Lett. 2012;315–316:30–40.

20. Bouttes N, Lhardy F, Quiquet A, Paillard D, Goosse H, Roche DM. Deglacial climate changes as forced by ice sheet reconstructions [Internet]. 2022 Nov. Available from: https://egusphere.copernicus.org/preprints/2022/egusphere-2022-993/

21. Vrac M, Drobinski P, Merlo A, Herrmann M, Lavaysse C, Li L, et al. Dynamical and statistical downscaling of the French Mediterranean climate: Uncertainty assessment. Natural Hazards and Earth System Science. 2012;12(9):2769–84.

22. Hubert B, François L, Warnant P, Strivay D. Stochastic generation of meteorological variables and effects on global models of water and carbon cycles in vegetation and soils. J Hydrol (Amst). 1998;212–213:318–34.

23. Otto D, Rasse D, Kaplan J, Warnant P, François L. Biospheric carbon stocks reconstructed at the Last Glacial Maximum: Comparison between general circulation models using prescribed and computed sea surface temperatures. Glob Planet Change. 2002;33(1–2):117–38.

24. Giesecke T, Brewer S, Finsinger W, Leydet M, Bradshaw RHW. Patterns and dynamics of European vegetation change over the last 15,000 years. J Biogeogr. 2017;44(7):1441–56.

25. Svenning JC, Skov F. Limited filling of the potential range in European tree species. Ecol Lett. 2004;7(7):565–73.

26. Seliger BJ, McGill BJ, Svenning JC, Gill JL. Widespread underfilling of the potential ranges of North American trees. J Biogeogr. 2021;48(2):359–71.

27. Dallmeyer A, Kleinen T, Claussen M, Weitzel N, Cao X, Herzschuh U. The deglacial forest conundrum. Nat Commun. 2022;13.

28. Svenning JC, Sandel B. Disequilibrium vegetation dynamics under future climate change. Am J Bot. 2013;100(7):1266–86.

29. Birks HJB, Birks HH. Biological responses to rapid climate change at the Younger Dryas-Holocene transition at Kråkenes, western Norway. Holocene. 2008;18(1).
